# An apple a day – how the platform economy impacts value creation in the healthcare market

**DOI:** 10.1007/s12525-021-00467-2

**Published:** 2021-04-14

**Authors:** Alexander Gleiss, Marco Kohlhagen, Key Pousttchi

**Affiliations:** 1 Chair for Business Informatics, University of Potsdam, August-Bebel-Str. 89, 14482 Potsdam, Germany; 2 wi-mobile Prof. Pousttchi GmbH, Birkenstr. 1, 14469 Potsdam, Germany

**Keywords:** Digital platforms, Platform economy, Healthcare market, Digital health, GAFAM, Value network analysis, I11, L14, L22, M13

## Abstract

The healthcare industry has been slow to adopt new technologies and practices. However, digital and data-enabled innovations diffuse the market, and the COVID-19 pandemic has recently emphasized the necessity of a fundamental digital transformation. Available research indicates the relevance of digital platforms in this process but has not studied their economic impact to date. In view of this research gap and the social and economic relevance of healthcare, we explore how digital platforms might affect value creation in this market with a particular focus on Google, Apple, Facebook, Amazon, and Microsoft (GAFAM). We rely on value network analyses to examine how GAFAM platforms introduce new value-creating roles and mechanisms in healthcare through their manifold products and services. Hereupon, we examine the GAFAM-impact on healthcare by scrutinizing the facilitators, activities, and effects. Our analyses show how GAFAM platforms multifacetedly untie conventional relationships and transform value creation structures in the healthcare market.

**Supplementary Information:**

The online version contains supplementary material available at 10.1007/s12525-021-00467-2.

## Introduction

Apple CEO Tim Cook predicted in 2019: *“I believe, if you zoom out into the future, and you look back, and you ask the question, ‘What was Apple’s greatest contribution to mankind?’ it will be about health”*, The other GAFAM (Google, Apple, Facebook, Amazon, Microsoft) platforms have already placed similarly visionary statements about healthcare. This sector is not only relevant to GAFAM platforms, but also of vital importance for both society and economy. Global spending on health amounted to US$ 7,8 trillion in 2017 and continues to rise, partly because of expensive digital technologies (WHO 2019). Recently, the COVID-19 pandemic has yet again elevated the importance of digital health solutions. As a result, advanced digital and data-enabled technologies increasingly diffuse the healthcare market, which undergoes a costly and massive digital transformation (Agarwal et al. 2010; Lapāo 2019; Menvielle et al. 2017; Pousttchi et al. 2019).

Both academic and practical literature indicates that digital platforms might decisively contribute to that transformation (Chen 2019; Hermes et al. 2020; Zenooz and Fox 2019). Digital platforms provide infrastructures to either facilitate transaction or collaborative innovation among complementary user groups (Hein et al. 2020). Big digital platforms have concentrated enormous power and radically changed our work, private and social lives. This particularly accounts for Google, Apple, Facebook, Amazon, and Microsoft, as the most successful platforms and most valuable companies in the world (van der Aalst et al. 2019). These players build upon personal data that consumers produce with their services or products, and employ these data to create personalized services or offer pinpoint advertising space to other companies. Health-related data might become another puzzle piece to complete the big picture, and recent GAFAM activities and statements underpin their ambitions strongly (Kimmell 2019b).

Available research has examined digital platforms from various perspectives and disciplinary approaches (Abdelkafi et al. 2019; Sutherland and Jarrahi 2018). However, there is comparatively little knowledge and theoretical conceptualization from a holistic standpoint on how and to what extent big digital platforms redesign entire ecosystems and markets (Asadullah et al. 2018; Hermes et al. 2020). In particular, research has not studied the potential strategic economic and technological impact of the big digital platforms on healthcare. This is remarkable, given the economic and societal importance of healthcare and the tremendous weight of platforms in other industries (Kenney and Zysman 2016). The platform impact on healthcare has not become fully visible to date, but European attempts to access Apple’s and Google’s Bluetooth APIs to monitor COVID-19 infection chains have recently revealed the platform-dependency even of entire states (Vincent 2020). Stories like this typify the increasing importance of digital health in general and of GAFAM platforms in healthcare in particular, regardless of their potential benefits or risks for patients, healthcare market incumbents, and the society at large.

Against this background, we aim to explore the potential economic impact of digital platforms in the vibrant healthcare market. To limit the scope of our analysis, we focus on GAFAM platforms to examine how they affect conventional value creation structures in the healthcare market. Thus, this study contributes to a deeper understanding of how big digital platforms entangle entire markets. We approach our study from a strategic management perspective and go beyond value chains to analyze value creation through intersectoral value networks with several value-adding parts that eventually culminate into an overall value proposition in the form of final goods or services for end-users (Bowman and Ambrosini, 2000; Mol et al. 2005; Pagani 2013).

At this, we rely on value network analyses of the healthcare market to explore how GAFAM services and products induce new value-creating roles and mechanisms in healthcare. Hereupon, we examine the GAFAM-impact on healthcare by scrutinizing the facilitators, activities, and effects. The rest of this paper is structured as follows: Next, we provide background information on digital platforms in healthcare and our methodical approach. Upon this, we conduct value network analyses of both the conventional and platform-induced healthcare market to analyze and discuss the GAFAM-platform impact on healthcare. We conclude with practical and theoretical implications of our contribution and provide avenues for future research.

## Background

### Theories on digital platforms

To explore the potential impact of GAFAM platforms on the healthcare market, we need to understand the nature of digital platforms. Research on digital platforms has obtained broad coverage in IS and Economics within the past decade (de Reuver et al. 2018; Hagiu and Wright 2015; Sriram et al. 2015; Willing et al. 2017), as the progress of digital technologies has elevated the impact of platforms considerably (Parker et al. 2016).

According to theory, all platforms exhibit two fundamental characteristics: They facilitate (1) *direct interaction* between two or more distinct sides of user groups by reducing transaction costs. Hence, platforms can control the transaction’s key terms, e.g., pricing of goods, or program language. All user groups maintain an (2) *affiliation* with the platform. Hence, they make platform-related investments to engage in the interaction (Hagiu and Wright 2015). These characteristics specifically apply to big digital platforms like GAFAM that draw on large user bases and enable interaction among several user groups, which makes them capable of integrating (formerly separated) markets both horizontally and vertically (Galloway 2018, p. 186; van der Aalst et al. 2019, p. 646). So far, GAFAM platforms have largely exploited their power in consumer markets (B2C) by connecting companies (e.g., product, service, and content providers) with end-users. However, they increasingly expand to business-to-business markets (B2B), which are still rather fragmented (Dolata 2017; Hein et al. 2020), such as healthcare.

Existent theory holds two further platform-specific characteristics: (3) *Network effects*, which arise from bringing together similar or complementary user groups (Parker and van Alstyne 2005; Rochet and Tirole 2003). Thus, a platform’s usefulness (and therefore value) is subject to the size of relevant participants (Eisenmann et al. 2006; Shapiro and Varian 1998). (4) *Homing and switching costs* incur for participants due to platform affiliation (Armstrong 2006; Evans et al. 2006; Kwon et al. 2017). Particularly in B2C, GAFAM platforms have carried these effects to the extreme; many companies and consumers are bound to these digital ecosystems (Bender 2020). Especially consumers rely on digital devices and channels, either to connect to the digital world (Apple, Google), maintain social relationships (Facebook), go shopping (Amazon), or be productive (Apple, Microsoft) (Baumöl et al. 2016; Pousttchi and Dehnert 2018). It stands to reason that consumers might adopt such habits or ties as patients.

Available research has yet conceptualized different types of digital platforms. Gawer (2014) provides three classes concerning a platform’s orientation and sphere of activity: internal platforms (of one company and its sub-units), supply-chain platforms (for assemblers and suppliers), and industry platforms (of a platform owner and complementors). While the first two types hold limited access and innovative capabilities, the third type provides open interfaces for a potentially unlimited pool of external capabilities. Google, Apple, and Facebook represent this third type par excellence (Gawer 2014). Other research differentiates digital platforms by their purpose: transaction, innovation, integrated (or hybrid) (Abdelkafi et al. 2019; Evans and Gawer 2016). While transaction platforms facilitate exchange between different groups, innovation platforms serve as a foundation on top of which groups can develop complementary or additional technologies, products or services. Integrated platforms combine both features into a more powerful platform type. Depending on the particular service, GAFAM platforms can be assigned to all platform types, although their core services represent the most powerful category of integrated platforms (Evans and Gawer 2016).

Irrespective of the platform type, there are three building blocks of digital platforms and their ecosystems: *platform owners* and the degree of power centralization, *platform complementors* with their contributions and autonomy, and *platform value-creating mechanisms* for facilitating transactions and innovation (Hein et al. 2020). Thus, GAFAM platforms are reliant on complementary actors. This entails in healthcare, among others, patients, physicians, hospitals, and other service providers to create value. The fragmented healthcare market consequently offers business and growth opportunities to GAFAM platforms to leverage their value-creating mechanisms. Especially, market-oriented research exhibits that digital platforms have proven to provide successful business models (Abdelkafi et al. 2019) which are able to transform and burst formerly grown value chains, existing competitive structures, and entire markets (Alt and Zimmermann 2019; Pousttchi and Gleiss 2019).

### Digital transformation and GAFAM in healthcare

The healthcare sector has moved into the digital world with some delay. On an organizational level, healthcare providers have been slow to adopt new technologies and have relied on paper-based processes for a long time (Agarwal et al. 2010). On a structural level, high costs, complex regulatory systems, and missing standards have impeded digital progress (Otto and Harst 2019). However, the healthcare sector faces an expensive and massive digital transformation (Burton-Jones et al. 2020) owing to new digital technologies and personal expectations (Menvielle et al. 2017; Safavi and Kalis 2020). Most recently, the COVID-19 pandemic has stimulated the need for digital health (Fagherazzi et al. 2020). The current progress in both research and practice underpin this impression strongly (Agarwal et al. 2010; Raghupathi and Tan 2008; Vogel et al. 2013; Wickramasinghe and Kirn 2013). Most of all, digital technologies make the promise to improve efficiency and communication, optimize or personalize medical treatment, support decision-making, empower staff, or remove boundaries, due to advances in data analytics and artificial intelligence (AI), (medical) internet of things (IoT), mobile health (mHealth) and virtualization (Dimitrov 2016; Gopal et al. 2019; Raghupathi and Raghupathi 2014; Safavi and Kalis 2020).

To date, the digital health ecosystem has several incumbent players that develop information and medical technologies for healthcare providers, including large companies like IBM, Cerner, AllScripts, or athenahealth (Correa 2020). Around these players, the market is quite scattered with thousands of small, highly specialized firms and startups (Cohen et al. 2020). The segments for hospital information systems (HIS) and electronic health records (EHR) are matured, and the big incumbents seek new segments based on other health IT solutions, especially in terms of networking, data analytics, and virtual services (Dyrda 2020; Gregg 2015). Practical evidence indicates that digital platforms might also play an important part in the digital transformation in healthcare (Chen 2019; Kuchler 2020; Pearl 2019; Zenooz and Fox 2019).

First, the market is highly fragmented, both on-demand and supply side, with many potential user groups to intermediate (e.g., healthcare providers, patients, insurers, digital health solution providers). Digital platforms might facilitate interaction among these user groups. Especially, GAFAM platforms have proven to be efficient matchmakers. Second, the digital transformation is just gathering speed, both government-driven (top-down) and consumer-driven (bottom-up). Governments might foster innovation platforms and the establishment of standards, and GAFAM platforms have proven that they can comply with healthcare industry and regulatory standards such as HIPAA (Health Insurance Portability and Accountability Act) or FHIR (fast healthcare interoperability resources) (Barbier-Feraud et al. 2016; Jindal 2019). Anyway, it is difficult to offer digital-health solutions to patients without employing the ecosystems and infrastructures of GAFAM. What is more, they possess both the required technological and economic capabilities to occupy an important part in this transformation process.

Third, digital health is a global growth market. There are several incumbent health and medical IT companies (e.g., Cerner, Allscripts, Medtronic), but in view of the emergent digital transformation, the claims are not staked and new players might enter the market. GAFAM platforms consider the healthcare market as a favorable opportunity for expanding their economic power and data pools (Chen 2019; Kuchler 2020; Pearl 2019; Zenooz and Fox 2019). They are already involved in healthcare in many ways. For instance, Apple and Google have entered into a rare partnership to co-develop a Bluetooth-based technology that facilitates contact tracing during the COVID-19 pandemic, and Facebook and Microsoft are initial members of the Public Health Tech Initiative (PHTI) from the Consumer Technology Association to explore digital solutions for future pandemics. As a basis for further analysis, we aim to describe and examine the actual business activities in healthcare of each GAFAM platform separately.

**Google** (or its mother company Alphabet, respectively) has gained its power basically from two platform ecosystems with tremendous market shares: Google Search (92%) with its compliant services (e.g., Maps, Mail, Analytics, Chrome), and the mobile operating system (mOS) Android (76%) as collaborative infrastructure for mobile devices and services (StatCounter 2020a, b). Accordingly, Google has gained enormous ICT capabilities, particularly in terms of intermediation, marketing, IT service design and, most of all, data analytics and AI (Galloway 2018). The healthcare market is seen to be another growth area for Google to play off these assets (CB Insights 2018; Google Health 2020). Today, Google has already several direct and indirect stakes in the healthcare market by focusing on its ICT core competencies. Google with its *Search* engine and *Assistant* is oftentimes the first contact point for patients when gathering information about symptoms or looking for physicians (Drees 2019). The tools provide findings from content created by medical experts and validated by Google (Gibbs 2015). *Android OS* (including *Google Play*) provides smartphone users access to several health-related mobile apps from third-party developers (and vice versa). Plus, Google offers *Android Things* as OS foundation for IoT devices and has recently launched *Google Fit* which helps users to monitor their activity data and wellbeing through connected wearables (e.g., *Fitbit*). All these services may support Google to approach the healthcare market from B2C.

In B2B, the company had initiated *Google Health* in 2008, an electronic PHR (personal health record) platform, but stopped this project in 2012 owing to a lack of demand and regulatory issues. Instead, Google currently develops a new *EHR tool* (Matthews 2020). Google also provides *Workspace* to healthcare providers, which is a cloud and groupware solution combining several tools for data management and collaboration. To support such solutions, Google bought *Apigee* in 2016 to provide APIs which are compatible with relevant healthcare standards. Plus, the smart home portfolio of *Nest* can help healthcare providers to observe their patients. Furthermore, Google invested a lot of money in research-oriented products and projects, such as *Cloud Life Services*, a genome database and collaboration platform for life and data science. Hence, Google laid the foundations to provide platform services in the B2B healthcare market.

Among Alphabet’s subsidiaries, at least four projects are already active in the healthcare sector: *Verily Life Science* kickstarted a series of promising research projects from smart contact lenses to reactive cutlery for Parkinson’s patients, *Calico* strives to unravel the mystery of human aging processes, *DeepMind* develops AI systems for automated diagnosis tools, and *Wing* shall revolutionize transportation with drones, which also might convey pharmacies, specimen or donor organs. So far, these activities might be rather of symbolic importance but underline the strategic ambitions of Google to play to their strengths and explore new business opportunities.

**Apple**’s success primarily stems from providing an all-in-one and well-matched digital ecosystem consisting of devices, systems, and services (Galloway 2018). However, the consumer electronics market approximates saturation, and new revenue sources are sought (e.g., subscription services like Apple One). The healthcare market offers three major opportunities for Apple: (1) selling more devices, (2) selling digital health services, and (3) exploit new data sources (Coldewey et al. 2020; Kimmell 2019a).

To date, Apple has several points of contact in healthcare. From a B2C perspective, *iOS* users can download several health-related apps from the *App Store* to their *iPhones*. The *Health App* is already preinstalled and allows its users to track, trace, and analyze their activity- and body-related data from smartphones or wearable sensors, such as ECG data from the *Apple Watch*. Plus, iOS users might ask *Siri* about their health-related issues. Apple has also developed and introduced platform solutions for B2B. This includes *Health Records*, a tool for patients to share health data with healthcare providers. This ecosystem is completed by *HealthKit*, a central repository and framework for developers to manage and merge data from multiple sources. Additionally, the software frameworks *ResearchKit* and *CareKit* support the development of apps for clinical studies and the monitoring of health-related data. Mobile and smart devices like the *iPad* or *HomePod* could support medical care staff or researchers. Apple even erected two employee-exclusive health clinics close to the headquarter in 2018, *AC Wellness*, which provide a suitable environment for the development and testing of new devices or digital health solutions.

**Facebook** is the predominant social media ecosystem in the Western society. Thus, Facebook knows about the communication and interests of billions of users. After the company tried in vain to develop own mobile devices or launch an own mobile operating system, it mainly concentrated on marketing (Galloway 2018). However, the healthcare market might be seen as a new playground to catch up with Google or Apple. Here, Facebook particularly concentrates on advances in AI and virtualization.

Facebook’s activity range in healthcare is comparatively narrow but yet impactful. For instance, (especially chronically ill) patients refer to social media such as *Facebook* or *WhatsApp* to communicate with physicians or fellow sufferers (Househ et al. 2014). Likewise, WhatsApp is widely used among healthcare professionals in hospitals (De Benedictis et al. 2019). Data from their social network services help Facebook to promote AI-based projects (e.g., for suicide prevention). The initiatives of Facebook’s AI research institute FAIR include the *fastMRI* project with the NYU School of Medicine to improve and accelerate radiological techniques. In terms of virtualization, Facebook operates its Reality Labs consisting of five research facilities to develop new solutions based on virtual and augmented reality (VR/AR). Exemplarily, the *CTRL-kit* provides a non-invasive neural interface platform that allows for new types of human-computer interaction (HCI). Moreover, the *Oculus* VR glasses have proven useful for educational purposes in medicine and surgery.

**Amazon** is the most successful marketplace platform in the Western world (Galloway 2018), and while many companies will not survive the COVID-19 pandemic, Amazon could even capitalize on the crisis (Semuels 2020). And although the marketplace platform generates the greater part of Amazon’s revenue, its cloud solutions account for the big profits (Chan 2020). Therefore, the healthcare market is not only another marketplace for Amazon but also an opportunity to demonstrate their capabilities in logistics and ICT (infrastructure, cloud, data analytics).

Amazon captures the healthcare market from several sides by exploiting its economic and technological capabilities. First, Amazon offers its market-leading cloud solution *Amazon Web Service (AWS)* to participants in healthcare and provides the AWS tool *Comprehend Medical*, which is a natural language processing (NLP) service to extract relevant medical information from unstructured text. Second, Amazon plays to its strengths in B2B and B2C commerce as both *Amazon Business* and *Amazon Marketplace* offer a broad range of medical devices and products, and control the delivery processes (*Amazon Logistics*).

Complementarily, Amazon launched *Basic Care* in 2017, an own brand for over-the-counter (OTC) medicine, and acquired *PillPack* in 2018, an online pharmacy which specialized in personalized, pre-sorted drug blisters. Third, Amazon has successively expanded the *Echo* and *Alexa Assistant* ecosystem by health-related services and applications through digital-health startup cooperations (e.g., *Livongo*). To explore new horizons, Amazon has pursued the health insurance project *Haven*, which was a joint venture with Berkshire Hathaway and JPMorgan Chase. Moreover, Amazon has launched a virtual medical clinic for employees in 2019, *Amazon Care*.

**Microsoft** has its core assets in the provision of software and cloud-based solutions (e.g., *Windows, Office*) for consumers and companies, but is virtually non-existent in the mobile world (Enderle 2019). Hence, the healthcare market might be seen as an opportunity to gain ground on the other big platforms (especially, Google and Apple). At this, Microsoft strives to exploit its technological capabilities to enhance public health. Microsoft’s initiative *Healthcare NExT* focuses on the support of healthcare innovations with AI and cloud computing, such as the *Healthcare Bot* for automated first-contact communication. The bot service is available via Microsoft’s *Azure Cloud*, which also complies with FHIR.

Just like Google, Microsoft has recently shelved its PHR platform *HealthVault*. Instead, the company provides its patient-centered EHR solution *ehCOS EHR* but mainly focuses on bringing its collaboration services *Microsoft 365* (incl. Office) and *Teams* to the healthcare market. Concerning hardware, the *Surface* line-up (incl. Windows and 365) shall address the need for portability in healthcare. Additionally, Microsoft has developed its *HoloLens* (i.e., AR glasses) with several possible applications in healthcare such as real-time ultrasound simulations or virtual training lessons for medical staff.

**Fig. 1:**
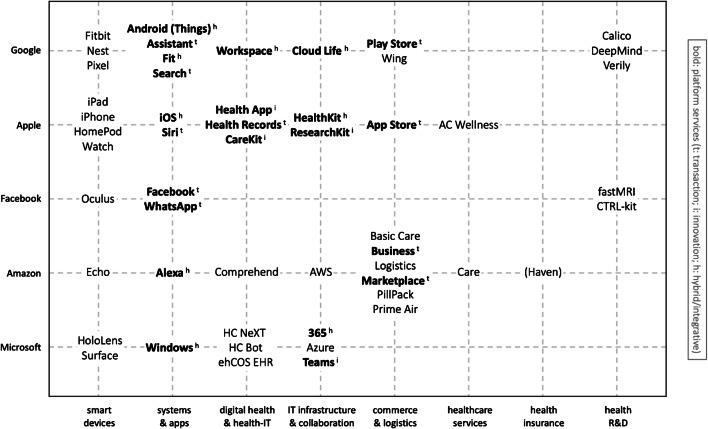
GAFAM activities in the healthcare market

Altogether, GAFAM platforms already pursue manifold activities in healthcare, even though the market is highly regulated. Many activities do not appear to be platform-specific or core-business related on their face. However, they help GAFAM platforms to collect data for providing and brokering personalized services, and thus contribute to their overarching strategies. Apart from that, the mere range and scope of GAFAM activities give reason to analyze their impact on the socially and economically important healthcare sector. For one thing, they exhibit the necessary financial and technological capabilities to develop digital health solutions or acquire those through mergers and acquisitions (M&A). Particularly, this applies to (big) data analytics and AI, cloud infrastructures and services, virtualization of products and services, and new forms of HCI. For another thing, GAFAM platforms can build upon their existing consumer relations and proximity to capitalize on the patient-driven part of the digital transformation in healthcare. Thus, they might throw a bridge from B2C to B2B. Figure 1 summarizes the healthcare-related business activities from all five GAFAM platforms, and illustrates in what sub- and cross-segments they are already active in healthcare.

### Literature review: Digital platforms in healthcare

Big digital platforms, especially GAFAM, play a crucial role in our everyday lives and have powerful implications for society and economy (Alt and Zimmermann 2019; Kenney and Zysman 2016; van der Aalst et al. 2019). However, in light of the ongoing massive digital transformation in healthcare and the insinuated platform-related developments, comparatively few contributions within IS have yet explored the potential impact of digital platforms on the healthcare market. Basically, available research on digital platforms explores the phenomenon from three perspectives (Hein et al. 2020): While the *market-based perspective* pays attention to the economic dimension of digital platforms (Tan et al. 2015), the *technological perspective* regards digital platforms as an extensible codebase of software-based systems providing a core functionality with complementary modular services which interoperate through shared interfaces (Tiwana et al. 2010). At last, the *user-centric perspective* concerns platform government mechanisms and actor relations.

From a *market-based perspective*, the demand side of healthcare platforms can be broadly subdivided into three user groups, i.e., patients, healthcare service providers, and payers. Platforms do not only interconnect these three parties with each other, but also with companies on the supply side which offer products, services, or digital applications (Fürstenau and Auschra 2016). Upon this, Fürstenau et al. (2019) developed a platform management framework to understand the interdependencies of a healthcare provider-led platform. The study allows the inference that incumbent market players in healthcare (e.g., hospitals or ICT providers) might struggle to successfully provide digital platform solutions for the entire healthcare sector, which affords big tech players a great opportunity to enter the market. Particularly, digital platforms might successfully address two main problems in healthcare – namely, high fragmentation and low innovation – by enabling shared data repositories, interoperability, and the integration and innovation of new services (Fürstenau et al. 2019). Recently, Hermes et al. (2020) have investigated the digital transformation of the healthcare industry by analyzing 1830 healthcare organizations. Their study revealed technology-induced shifts in value creation in healthcare, resulting in new market segments, roles, and value streams. Digital platforms contribute to that shift, which gives reason to explore the GAFAM impact in-depth and in greater detail.

From a *technological perspective*, the healthcare system could profit from platform openness like no other industry, both in a technical and semantic sense (Estrin and Sim 2010), which implies issues like interoperability of technical standards as well as the provision of data from all stakeholders. More specifically, Vesselkov et al. (2019) focused on data production and consumption at mobile health platforms. Important aspects include data scope, data sharing, platform design, and platform governance, which all represent core assets of GAFAM companies. Generally, there is a need for technological progress in healthcare, particularly in terms of information infrastructures and applications to facilitate intra- and inter-organizational collaboration (Aanestad et al. 2019).

From a *user-centric perspective*, some contributions have already addressed the emergence of large digital platforms in the healthcare market. Gu and Hong (2019) explored the dissemination of health misinformation on social media apps. As the use of online social networks affects health-related behaviors, this has a lasting impact on the wellbeing and health status of their users (Durst et al. 2013). Kuebel et al. (2015) examined the adoption of smart home platforms and emphasized the importance of complementary assets like services, infrastructures, or brand image. Further research is rather patient-centric and levels out on the issues of PHRs, health and fitness apps, social media use, or specialized platforms which provide services for communication, information, diagnosis, or treatments (Bandyopadhyay et al. 2012; Huang et al. 2018; Kordzadeh and Warren 2017; Liu et al. 2020; Schaarschmidt et al. 2017; Zhang et al. 2019).

Altogether, we find a vast amount of general literature on the platform economy (Sutherland and Jarrahi 2018; Hein et al. 2020), growing research interest in this area (Alt and Zimmermann 2019), and the undeniable social, economic and technological impact of (particularly) big digital platforms (van der Aalst et al. 2019; Kenney and Zysman 2016). The presented literature provides several opportunities for GAFAM platforms to challenge the healthcare market (see Table 1). However, there is yet surprisingly little research that conceptualizes how digital platforms transfer and leverage their genuine services, economic assets, and technological capabilities into the healthcare market. Such a holistic, strategic view on the impact on entire markets and historically evolved ecosystems is yet missing (Asadullah et al. 2018).

**Table 1:** GAFAM platform potentials in healthcare (literature review)

perspective	GAFAM platform potentials in healthcare	Reference
market	- need to connect different stakeholders - incumbents’ inability to provide meta-platforms - high fragmentation & low innovation - rise of new market segments & value streams	Fürstenau and Auschra 2016 Fürstenau et al. 2019 Fürstenau et al. 2019 Hermes et al. 2020
technology	- need for openness & interoperability - need for data & platform competences - need for information infrastructures & applications	Estrin and Sim 2010 Vesselkov et al. 2019 Aanestad et al. 2019
user	- increasing relevance of social media - increased relevance of personal health (apps) - diffusion of IoT and smart home technologies - relevance of personal health records - relevance of patient empowerment - relevance of online communities - relevance of digital services (e.g., consultation)	Gu and Hong 2019; Liu et al. 2020 Huang et al. 2018 Kuebel et al. 2015 Bandyopadhyay et al. 2012 Schaarschmidt et al. 2017 Kordzadeh and Warren 2017 Zhang et al. 2019

## Methodology

We address the research gap and aim to examine how GAFAM platforms affect value creation in the healthcare market. We rely on value network analysis, which is particularly suitable to analyze digitalized, networked market structures (Peppard and Rylander 2006) and the role of new entrants within (Christensen and Rosenbloom 1995). At this, a value network is a dynamic cluster of economic entities with distinct tasks and responsibilities, which collaboratively co-produce and deliver value for the end-consumer in terms of offerings (Pagani 2013; Lusch and Vargo 2006). Such collaborative co-production is represented by the exchange of such offerings (i.e., value linkages). This market-oriented perspective of value networks refers to the transitional concept from goods- to service-dominant logic that focuses on the successive value delivery of services and offerings and their dynamic co-production across value chains (Lusch and Vargo 2006).

**Fig. 2:**
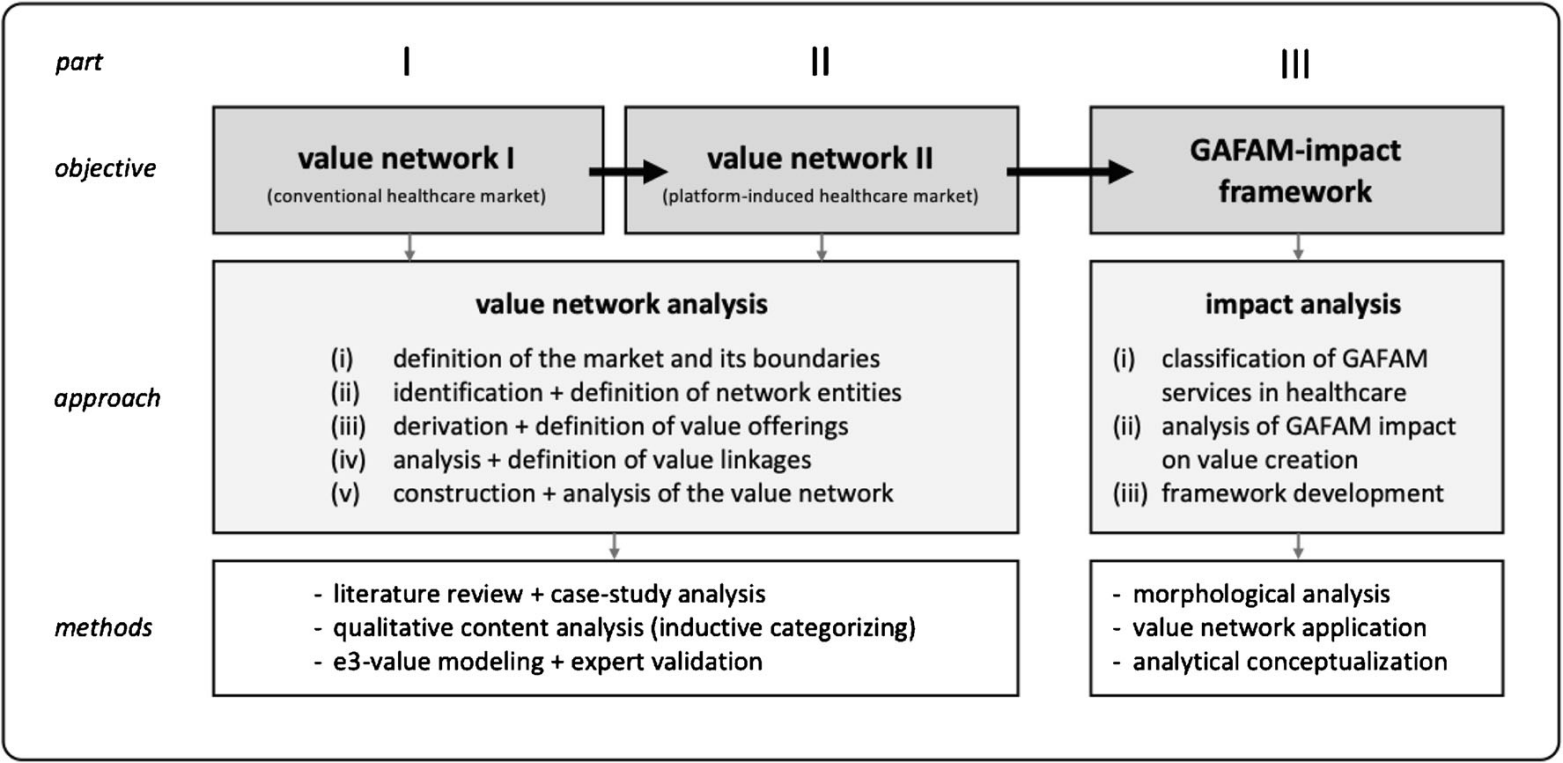
Research design

Our research design follows three superordinate parts: (I) Value network development and analysis of the *conventional healthcare market* to depict the initial situation without GAFAM platforms, (II) Value network development and analysis of the extended *platform-induced healthcare market* as systematic foundation for the illustration and examination of the platform-induced implications, and (III) Systematic derivation and analysis of the strategic impact of GAFAM platforms on value creation in the healthcare market, culminating into a *GAFAM-impact framework* (see Fig. 2).

Thus, part I and II imply the development of two value networks as a proper foundation to contrastingly analyze the GAFAM impact on the healthcare market. For the purpose of an appropriate value network development and analysis, we stick to the five-step process from Peppard and Rylander (2006). First, this includes the (i) definition of the market and its boundaries. In view of our research aim, we consider the European and U.S. healthcare market. However, we disregard the regulatory body to reduce complexity and enable generalizability as national regulation is very specific.

Further steps of the value network analysis imply the (ii) identification and definition of the network entities and (iii) their value offerings. Here, we applied an exploratory, qualitative-empirical approach to gain a deeper understanding of the phenomenon from a market-based perspective (Myers 1997; Orlikowski and Baroudi 1991). Regarding the conventional healthcare market, we initially conducted a literature review on value creation and value networks in healthcare to identify different entities like consumers, service or product providers, suppliers, and peripheral supporters (Basole and Rouse 2008). We scanned scientific databases for the search string “(health*) AND (value OR network)”. We complemented this by a multiple case-study analysis to empirically explore and contextualize further required entities until theoretical saturation, i.e., further cases do not produce further entities (Yin 2009). This includes the review of healthcare-specific directories and reports, which we retrieved through an online search. Regarding the platform-induced healthcare market, we identified new entities and value offerings from the GAFAM business activities in healthcare (as presented previously in the background section). Here we rely on information from the company websites, articles from newspapers or magazines, and industry reports, which we collected from extensive online research. In order to generalize and conceptualize our literature- and case-based findings to network entities and their value offerings, we rely on Mayring’s method of qualitative content analysis for inductive category development (2000).

The final steps of the value network analysis consist of the (iv) definition of the value linkages among the entities, and the (v) construction of the value networks. Using our conceptualized and categorized empirical findings, we apply a design-science oriented approach to construct the two value networks. Based on practice- or theory-based insights, design science research (DSR) allows the generation of new knowledge through the design of novel artifacts such as models, concepts, or constructs (Hevner et al. 2004; March and Storey 2008; Vaishnavi et al. 2019). Following these principles, we apply a specific modeling method for the construction and representation of value networks. Our method basically rests upon e3-value, a systematic modeling approach to illustrate and analyze the value linkages among actors in business models (Gordijn et al. 2000) and multi-actor value networks in digitalized service environments (Hotie and Gordijn 2019). At this, value-adding activities are aggregated and assigned to actors, and their exchanged value offerings (e.g., data, money, product, service) are represented by value flows (Pousttchi 2008; Gordijn et al. 2000). To provide more generalizability, we employ an extended version of e3-value (Pousttchi 2008). Given the dynamics of such complex markets, the entities within our value networks are not represented by actual market actors but by generic *roles*, which provide distinct value deliveries and aggregate several value-adding activities for exchange with other roles. For the purpose of analysis, actual market actors can be assigned to one or more of such roles (Pousttchi 2008; Pousttchi and Hufenbach 2014). For reasons of generalization and specialization, similar roles can be aggregated to meta-roles. Table 2 presents the notation elements.

**Table 2:**
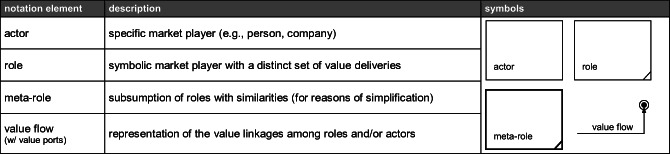
Notation of role-based e3-value modeling technique

Hence, our value networks fulfill the function of reference models. Reference modeling allows the inductive or deductive development of simplified or idealized system representations (Wilde and Hess 2007). In this context, our value networks do not represent a desired state, but support structuring and orientation for the purpose of universal applicability (Becker and Schütte 2004; Fettke and Loos 2007). Thus, both value networks can be considered as “abstract framework[s] for understanding significant relationships among the entities of some environment” (OASIS 2020), as in our case, roles representing entities within healthcare (Bernus 1999).

In order to assure the functional performance and suitability of our artifacts (Kuechler and Vaishnavi 2012), the value networks have been validated twofold: For one thing, we conducted an interdisciplinary workshop with academic researchers (from Informatics, BI, IS, Healthcare) at a digital health congress in 2019, which was organized by two special interest groups for digital health and mobile systems of the GI (German Informatics Society). For another thing, we conducted workshops and discussions with healthcare practitioners representing a broad range of healthcare actors: CIOs and Heads of IT from hospitals (i.e., *inpatient* healthcare), the board member of a regional association of statutory physicians (i.e., *outpatient* healthcare), and managers from regional healthcare cluster initiatives, health insurances, health-IT providers and health startups (i.e., *peripheral* healthcare actors). In the course of these activities, our value networks have been iteratively adjusted and refined.

These foundations support part III of our research process, i.e., analyzing the potential impact of GAFAM on value creation in healthcare. Here, we rely on the usefulness of reference models to generate theoretical and practical relevance in terms of description, explanation, and prediction (Fettke and Loos 2004; vom Brocke 2007; Gregor 2006). In this regard, the conventional healthcare market is of descriptive nature representing the status quo based on empirically derived roles from healthcare market actors. Contrastingly, the value network of the platform-induced healthcare market is rather hypothetical with an explanative and predictive nature, based on empirically derived and integrated roles from GAFAM business activities. Hence, the value networks aim to illustrate the differences owing to platform-induced value-offerings.

In order to analyze the resulting impact, we employ our reference models to systemize the GAFAM-related business models and to conceptualize the platform-induced impact on value creation in healthcare. Regarding the business models, we systematically decompound the platform-induced roles and value flows by employing the morphological analysis, a highly systematic method to structure multi-dimensional problems. It involves the identification and definition of the investigated phenomenon’s essential characteristics and the assignment of relevant instances to each characteristic. The aggregate of all critical characteristics and instances is represented by a morphological box, which allows for further analysis, systematization, and comparison of complex phenomena (Ritchey 2013; Zwicky 1966). Following these steps, we condensed the potential characteristics of GAFAM business models in healthcare into a morphological box. Based on these conceptual foundations, we analyze, theorize, and discuss the actual GAFAM impact on the healthcare market (Gregor 2006). We illustrate our analytical key inferences which culminate in a conceptual GAFAM-impact framework in healthcare.

## Value network of the conventional healthcare market

### Definition of conventional roles and value offerings

To conceive the impact of big digital platforms on value creation in healthcare, we need a basic understanding of how value is usually created and delivered in the healthcare market. Thus, we first develop the generic value network of the conventional healthcare market. Following the guidelines from Peppard and Rylander (2006), this entails the identification and definition of relevant value-creating and value offering entities in healthcare, and the value flows among them. The artifacts are inductively developed through a qualitative content analysis of healthcare literature and cases (Mayring 2000). Given our value network’s function as a reference model, the entities are represented by generic roles.

In a first approximation of value creation in healthcare, we refer to the central relationship between the *patient* who receives a healthcare service (e.g., curative or preventive medical treatment or information) from a physician, clinic, or hospital, which requires the role of a *medical care service provider* (SP). If the patient is uninsured, the service is charged to the patient’s account. If a healthcare service is covered by health insurance, it is either charged directly to the insurer’s account or reimbursable against receipt. Health insurance might be offered, e.g., by a public or private health insurance or maintenance organization from the employer, which is why we combine the affiliated tasks to the role of the *payer*. The payer usually defrays these expenses from insurance premiums from the shared-risk community. Thus, we basically find a triangle relationship between the *patient* (as service recipient), the *medical care SP* (as service provider), and the *payer*, which is common in many developed countries (Fürstenau and Auschra 2016).

Since the patient is seen as a final service recipient in our value networks, this entity is labeled as an actor. In view of the actual healthcare market, the roles of the payer and the medical care SP can be further partitioned. Depending on a patient’s insurance plan, healthcare treatment costs are either paid by the insurance company directly to the medical care SP (*Direct Cost-Takeover Payer*) or indirectly, i.e., the patient goes in advance and requests a return from the insurance (*Reimbursement-Oriented Payer*). This differentiation might become important in the next years with regard to process flexibility of reimbursing digital healthcare services (Gerke et al. 2020).

**Fig. 3:**
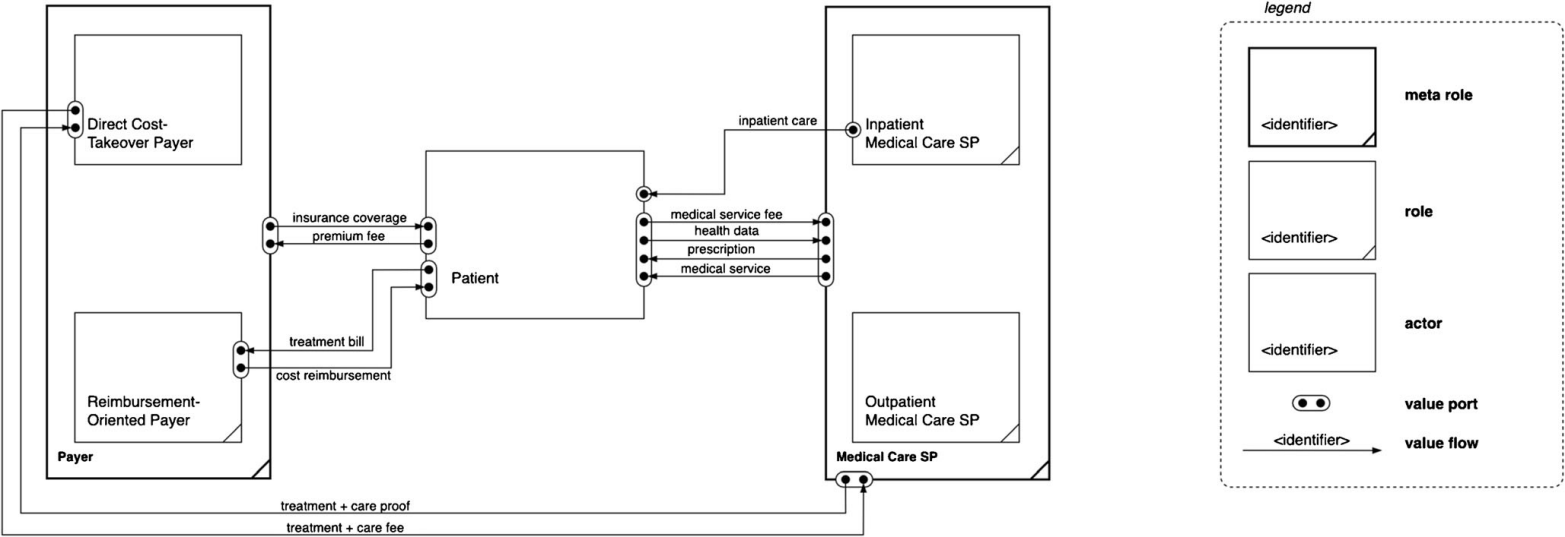
Value network excerpt of the healthcare market

In terms of the medical care SP, some healthcare services are ambulant (i.e., often acute, sporadic, or routine) and can be managed quickly (CDPH 2020; GoHealth 2017; Niemann and Burghardt 2016), while others require overnight stays and a longer treatment duration, which allows continuous monitoring of the health status and provides the possibility of immediate medical interventions. Hence, we differentiate between an *outpatient medical care SP* (e.g., registered physicians, clinics, or urgent care centers) that administers the ambulant medical care of day patients, and an *inpatient medical care SP* (e.g., hospitals) that processes the inpatient medical care of overnight patients who are either chronically ill or seriously injured on a temporary basis. Figure 3 depicts the value linkages among these roles through a value network excerpt based on e3-value. The roles of the payer and medical care SP are aggregated to meta roles to allow for generalization and specialization.

Both of these medical care SP roles carry out direct and indirect value-creating activities. Direct activities include medical services such as (preventive) consultation, information, diagnosis, and therapy, while indirect activities imply tasks such as procurement, patient administration, or internal logistics of patients and materials (Kawczynski and Taisch 2010; Myllärniemi and Helander 2012; Weissinger 2014). Sometimes, chronically ill or seriously injured patients who are incapable to live alone and need full-time care. The required role of an *inpatient nursing care SP* is usually assumed by (special) care homes. Similarly, an *outpatient nursing care SP* manages the ambulant nursing care of chronically ill people who suffer from permanent minor diseases and run their households, or seriously injured but convalescent patients. For the purpose of generalization, medical care SP and nursing care SP can be subsumed to a *healthcare SP*. Table 3 summarizes the identified and defined healthcare-related roles.

**Table 3:** Healthcare-related roles

Meta-	Role	Value delivery	Exemplary market actors
payer	direct cost-takeover payer	provision of insurance coverage (by direct cost transfer)	health insurance firms, like United Healthcare (USA), Allianz (EU), or Achmea (EU)
	reimbursement-oriented payer	provision of insurance coverage (claim approval based)	
healthcare SP	outpatient medical care SP	provision of ambulant medical care (e.g., diagnosis)	registered physicians
	inpatient medical care SP	provision of residential medical care (e.g., treatment)	hospitals
	outpatient nursing care SP	provision of ambulant nursing care (e.g., bathing services)	home-care companies
	inpatient nursing care SP	provision of residential nursing care (e.g., bathing services)	care homes

Professional healthcare services often rely on pathology tests from clinical specimens to obtain information about the health status of a patient and make evidence-based diagnoses. This requires further roles in the value network. For one thing, the role of the *medical laboratory SP*, which administers the tests and analyses, and a *laboratory courier SP*, which safely conveys both the specimen and reports with test results (Pinna et al. 2015; Walters and Jones 2001). Another important logistics-oriented role is the *patient transfer SP*, which carries patients under medical supervision from their homes or places of accidents to a healthcare SP. Another important contribution to value creation in healthcare is the production and provision of pharmaceuticals and medical devices (Pitta and Laric 2004), which requires additional roles in our network.

Accordingly, medical devices need to be produced and distributed, which involves five basic roles: *medical device manufacturer* and *medical device distributor*. At the distribution of drugs, we need to differentiate between drugs, which are subject to medical prescription, and those, which are obtainable over the counter (OTC drugs). Hence, we distinguish the roles of the *prescription-drug distributor* and the *OTC-drug distributor*. Both drug types are produced by the *drug manufacturer*. Since the shipping of pharmaceuticals is liable to severe restrictions and regulations, we propose the apposition of a *drug logistics SP*. Additionally, healthcare relies on continuous scientific progress and infrastructures, and therefore on the role of a pharmaceutical, biotechnological, medical, and technological *research SP* (see Table 4).

**Table 4:** Healthcare-support roles

Role	Description	Exemplary market actors
medical laboratory SP	provision of clinical (pathological) tests/analyses from specimens	Alere (USA), SynLab (EU)
laboratory courier SP	fast transfer of specimen and test results	LabLogistics (USA), GO! (EU)
patient transfer SP	emergency/safe transfer of patients from and to healthcare SP	MTM (USA), Green Cross (EU)
drug manufacturer	development and production of drugs and medicine	Pfizer (USA), Roche (CHE)
prescription-drug distributor	sales of prescription drugs to patients or healthcare SP	pharmacies: Walgreens (USA)
OTC-drug distributor	sales of OTC-drugs to patients or healthcare SP	drug stores: dm, (EU)
drug logistics SP	safe transfer of drugs and medicine to pharmacies or drugstores	Kühne+Nagel, DHL (EU)
med. Device manufacturer	development and production of medical devices	MedTron (USA), Fresenius (EU)
med. Device distributor	sales of medical devices	McKesson, Vitality (USA)
research SP	provision of research findings and infrastructures	specialized R&D companies

In order to facilitate a comprehensive treatment across different healthcare SP (with different specializations), the provision of an adequate ICT infrastructure is crucial (Bharadwaj et al. 2013; Kagermann 2015; Myreteg 2015). Hence, further roles are required in the value network. On the side of the healthcare service providers, this involves specialized health information and administration systems which ideally (1) orchestrate and support internal processes and organization, (2) allow the capture, import, storage and export of treatment data, (3) provide specialized clinical or medical IT applications and (4) enable the exchange of information and communication data with other stakeholders in the value network (Agarwal et al. 2010; Schlichter et al. 2014). For the provision and maintenance of such systems and applications, we propose the role of the *Health-IT SP*, as conventional hospitals run hundreds of IT systems and applications simultaneously. As many of these applications are not operated on-premise (Kaletsch and Sunyaev 2011; Schneider and Sunyaev 2015), we propose the role of a *cloud SP* to flexibly hold required storage and computing capacities available. For processes of billing and communication between payers and medical care SP, the market actors rely on EHRs that enable the storage of patient-related data and their sharing among healthcare providers and insurance companies (Blechman et al. 2012; Dehling and Sunyaev 2014). Thus, we introduce the role of an *EHR operator*. Table 5 summarizes the identified and developed ICT infrastructure roles in healthcare.

**Table 5:** ICT infrastructure roles

Role	Description	Exemplary market actors
health-IT SP	provision of specialized healthcare systems and applications	Cerner (USA), Dedalus (EU)
cloud SP	provision of flexible storage and computing capacities	IBM (USA), Telekom (EU)
EHR operator	storage, processing and transfer of clinical health records	GE, Epic, AllScripts (USA)

### Construction of the conventional healthcare market value network

Based on the derived roles, we apply value flow analysis and the extended e3-value modeling method to develop a reference model of the healthcare market value network. Thus, we derive and define the relevant value linkages among the roles which serve as foundation for the value flows, and therefore the construction of the value network. The basic value exchange in healthcare is constituted by the value linkage between patient and medical care SP. Uninsured patients have to bear such costs themselves. Insured patients can refer to their payer which pays for the service (directly or by reimbursement). Thus, we find several value linkages among the entities: For a proper, preventive or therapeutic, treatment, both patient and medical care SP rely on supporting and peripheric products and services. Patients demand drugs, medical devices, transfer services, or information, which are either self-paid or covered by the payer’s benefits (depending on the healthcare service and the patient’s insurance status). A payer pools the risks of all patients and has to equalize all health expenses with the total amount of insurance premiums. Table 6 exhibits exemplary value linkages as foundation for the value network.

**Table 6:** Excerpt of defined value linkages in the conventional healthcare market value network

From role/actor	Value linkage	To role/actor
patient	service fee; data (e.g., health information, insurance proof)	medical care SP
medical care SP	medical treatment or health service	patient
medical care SP	treatment proof	payer
payer	fee/reimbursement for patient treatment	medical care SP

Similarly, a medical care SP (e.g., hospital) demands drugs for its value-adding activities (e.g., diagnosis, treatment, and monitoring), which is bought at a drug distributor (e.g., pharmacy). Additionally, the purchase and implementation of ICT components and services aim to support and optimize the provision of healthcare services along the entire healthcare cycle and from admission to dismission. To operate the facility economically, a medical care SP needs to factor in such costs to calculate the prices of their treatment services or – if such prices are subject to regulation – make their processes more profitable either way. Suppliers of drugs, medical devices, or ICT solutions are reliant upon upstream service or goods providers to create value themselves.

**Fig. 4:**
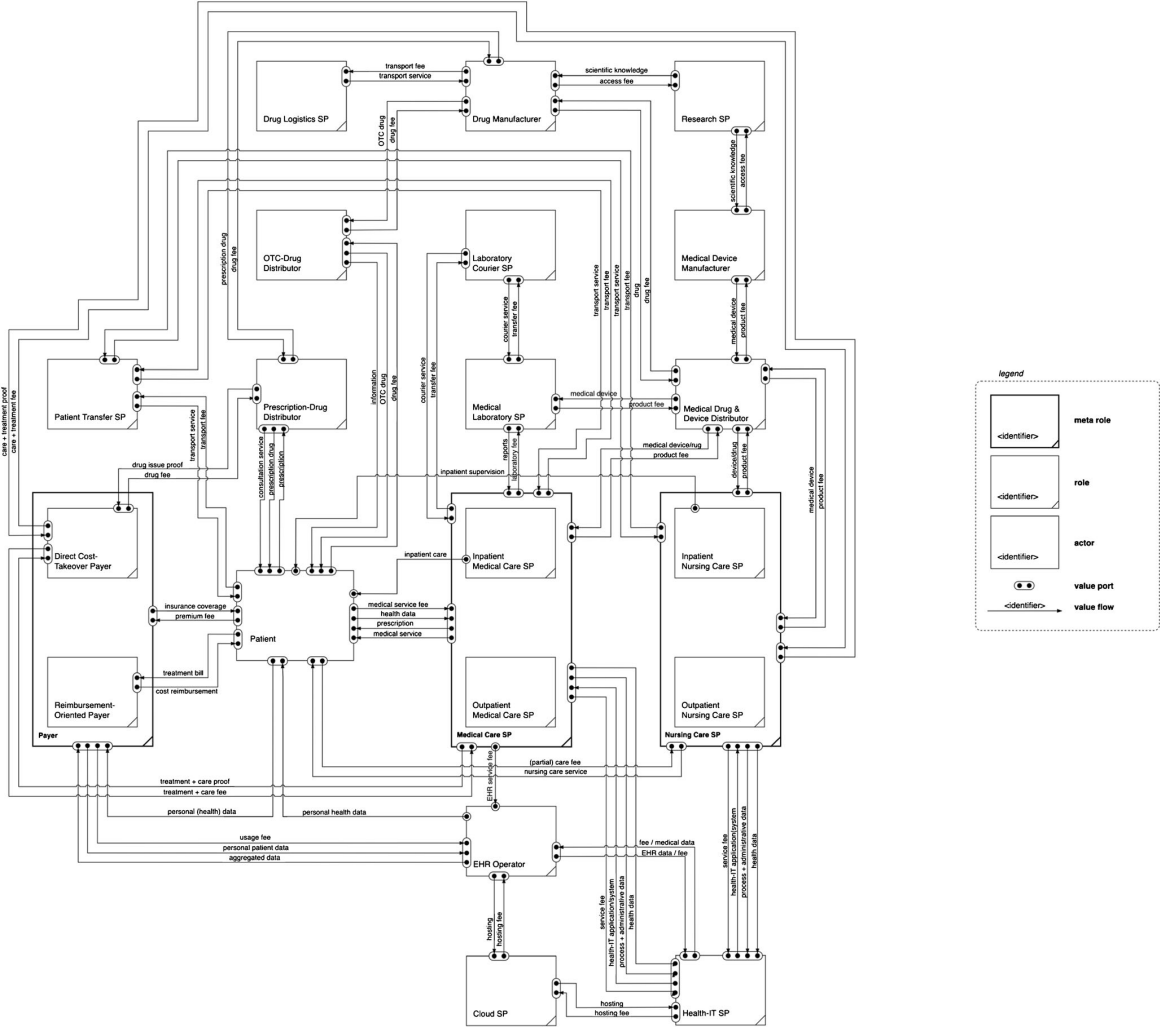
Conventional healthcare market value network

Through carefully connecting all these value linkages by value flows among the entities, we are able to construct a resulting role-based value network of the conventional healthcare market (see Fig. 4). Given its nature of a reference model, roles can be assigned to different real-world market actors depending on the considered scenario. For instance, a dentist (as outpatient medical care SP) could treat the patient using an endodontics system from the company Dentsply Sirona (as medical device manufacturer). Likewise, the value network can be used to analyze value flows of an infected patient who is carried by a patient transfer SP to the hospital (inpatient medical care SP), where the patient undergoes a screening, while the specimens are analyzed by an external medical laboratory SP. In view of our research aim and our following sections, the value network serves as foundation and starting point to analyze platform-induced changes in value creation in the healthcare market.

## Extension to a platform-induced value network

### Derivation and definition of platform-induced roles

In order to explore and understand how the value network of the healthcare market is affected by big digital platforms, we analyze the GAFAM business activities in healthcare presented in the background section. By means of a qualitative content analysis of the GAFAM business activities, we inductively derive and develop new platform-induced roles which affect the conventional value exchange in various ways, and thus value creation in the entire value network. As GAFAM activities concern different tie points in the value network, we sort and present these roles along with three inductively developed categories: patient, healthcare SP, and infrastructure.

### Patient-healthcare related roles

Several products and services of GAFAM platforms aim at directly fostering or supporting the patient’s healthcare processes. Some systems offer fast and easy access to (qualified or certified) health-related information or initial consultation (either automated or face-to-face). For instance, Google Search provides verified health content to certain health-related search queries and helps patients to quickly find, contact, or rate physicians in the vicinity. For such low-level and sporadic patient support, we propose the role of a *health orientation SP* which comprises activities such as the provision of health-related information, health system orientation, automated initial consultancy, or patient steering.

Other applications support the patient more continuously and provide services for data storing, monitoring, and analysis. Apple Health App combines personal health-related data from different apps to provide information on the individual health status. Likewise, similar applications facilitate peer communication rooms for exchanging experiences, as e.g., Facebook theme groups. Such services empower the patient to actively manage their health issues, which is why we propose the role of a *health assistant SP.* This role encompasses the provision of patient (decision) support for monitoring, analyzing, and sharing activity-related, physiologic, and medical data or feelings. Such services often require active commitment from the patient and demand their data for purposes of further processing. Other services, like Amazon Care, provide (direct or brokered) access to professional medical consultation from clinical staff. Such services more or less digitalize the traditional patient-physician relationship and have various manifestations. Thus, we propose the role of a *digital health SP* to provide medical services or content (either directly or brokered).

Likewise, Amazon enables patients to purchase both their required drugs and medical devices online from various suppliers, which is why we propose the roles of a *B2C drug marketplace* and *B2C medical device marketplace* to facilitate the provision of (brokered) drugs and medical devices, respectively. Amazon complements these services by a fast and convenient delivery of medical supplies for patients (Prime Air), which requires the role of a *drug delivery SP*. Table 7 summarizes all newly generated healthcare-related roles.

**Table 7:** New healthcare-related roles

Role	Value delivery	Exemplary GAFAM activity
health orientation SP	provision of health-related information, health system orientation, automated or personal initial consultancy, and patient steering	Apple Health App, Google Search, Facebook
health assistant SP	provision of patient (decision) support for monitoring, analyzing, sharing activity-related, physiologic, and medical data or feelings	Apple Health App, Facebook
digital health SP	provision of (brokered) professional medical consultation/content	Amazon Care
B2C drug marketplace	provision of (brokered) drugs and medicine for patient’s use	Amazon Marketplace
B2C medical device marketplace	provision of (brokered) medical devices for patient’s use	Amazon Marketplace
drug delivery SP	provision of quick supply of (OTC or prescription) drugs	Prime Air

### Healthcare SP support roles

The GAFAM platforms do not only provide products and services which target the patients. Many of their services rather focus on supporting the work of the healthcare SP, and thus on B2B. This requires further roles in the value network. Some services aim to optimize, automate, and facilitate clinical collaboration. For instance, Microsoft 365 has rolled out several tools and applications to support clinical staff in their daily tasks. To bundle such activities, we propose the role of a *groupware SP*, which comprises the provision of application systems with communication or collaboration tools for staff from healthcare SP. Other platform services support the government and monitoring of the healthcare SP’s IT landscape. For instance, Microsoft Azure Service Health provides a dashboard to monitor and control service updates and outages, planned maintenance, or issues from integrating other services or applications. Thus, we propose the role of an *IT Support Solutions SP* for the provision of application systems and tools to monitor, govern, and safeguard the operation of the clinical IT landscape.

Further platform services support clinical processes and decisions through automated documentation, processing, analysis, or sharing of data like, e.g., Amazon Comprehend Medical, an NLP service to extract relevant medical information from unstructured text. Such activities can be combined to the role of a *clinical support SP.* Additionally, GAFAM platforms provide holistic solutions for the provision of digital storage, exchange, and archiving of the patient’s entire medical records across payers, healthcare SP, and indications. For example, Apple Health Records enables hospitals to connect their EHRs to the patients’ mobile health apps for exchanging and aggregating data. Thus, we introduce the role of a *PHR operator* for the digital storage, exchange, and merging of a patient’s entire medical records.

Besides supporting IT services, GAFAM platforms also provide complementary technical devices based on advanced technologies. This includes the development of AR or VR glasses for improved diagnoses or surgery education, such as Facebook’s Oculus or Microsoft’s HoloLens. For such activities, we introduce the role of an *advanced devices provider*. Likewise, GAFAM platforms can help streamline the purchasing processes of healthcare SP. At this, new platforms like Amazon Business for ordering medical devices have risen recently, which requires the role of a *B2B medical device & drug marketplace* to ensure the provision of (brokered) medical devices and drugs for the healthcare SP’s use. Table 8 summarizes the defined healthcare-support roles.

**Table 8:** New healthcare-support roles

Role	Value delivery	Exemplary GAFAM activity
PHR operator	connector of digital storage, exchange and archiving of a patient’s entire medical records across patients, payers, healthcare SP	Apple Health Records
clinical support SP	provision of medical staff (decision) support through automated documentation, processing, analysis, or sharing of data	Amazon Comprehend Medical
groupware SP	provision of application systems with communication or collaboration tools for staff from healthcare SP	Microsoft 365 & Teams
IT support solutions SP	provision of application systems and tools to monitor, govern and safeguard the operation of the clinical IT landscape	Microsoft Azure Service Health
advanced devices provider	provision of advanced technological devices and applications for medical or educational purposes, e.g., AR/VR	Facebook Oculus, Microsoft HoloLens
B2B medical device & drug marketplace	provision of (brokered) medical devices for healthcare SP’s use	Amazon Business

### Infrastructure roles

GAFAM platforms comprise large technological capabilities to provide digital services and maintain the underlying infrastructures, especially in terms of data processing (Galloway 2018, p. 188; Petit 2016, p. 56). Hence, many platform-related business activities in healthcare focus on offering digital services, ecosystems, or infrastructures. In order to reach patients nowadays, healthcare SP rely on mobile devices and environments. Hence, the provision of mOS and smart devices is inevitable to bring services or content to the patient. Apple (iOS) and Google (Android) virtually form a duopoly for mOS, and thus have become a gatekeeper for any health-related mobile service. Thus, we propose the role of an *mOS provider* for the provision of digital infrastructure ecosystems (including frameworks, apps, and app stores), and the role of a *smart device provider* for the provision of connected cyber-physical systems (e.g., smartphones, wearables, smart home systems) with consumer interfaces to run the OS and corresponding applications mainly developed by third parties. In view of the IoT trend in healthcare, other data-collecting and processing with distinct OS might emerge and extend this role accordingly (e.g., Android Things, Nest).

However, mOS do not cover all patient-related data alone, since other applications or services (e.g., Amazon Marketplace, WhatsApp, or the Microsoft Healthcare Bot) collect and process a number of further health-relevant data, which need to be aggregated, stored, and prepared for further circulation and analysis. This requires the role of a *patient data & relationship SP* for patient communication and the collection, storage, and preparation of patient-related communication data from various devices, systems, and applications.

Some services have specialized in the standardization and harmonization of such data as well as the provision of suitable interfaces to allow a sharing of information among different stakeholders in the market. For instance, Apple Health App aggregates the patient health records from multiple institutions alongside their patient-generated data, which requires respective standardization procedures. Likewise, Microsoft provides an API to manage health-related data with Azure services. These activities require the role of a *data processing & exchange SP*. Many platform services comprehensively collect, store, prepare, and process large amounts of clinical and medical data sets for analytical purposes and rely on AI algorithms. For instance, Facebook’s FAIR initiative aims at supporting clinical IT solutions and processes with machine learning. Hence, we propose the role of an *AI support SP* for the provision of specialized health-related AI systems, applications, and algorithms. Table 9 summarizes all newly generated infrastructure roles.

**Table 9:** New infrastructure roles

Role	Value delivery	Exemplary GAFAM activity
mOS provider	provision of a data-processing infrastructure ecosystem, including frameworks, apps, services, and app stores	Alexa, iOS, Android
smart device provider	provision of connected cyber-physical systems with consumer interfaces, e.g., smartphones, wearables, smart home systems	Alexa, iPhone, Apple Watch
patient data & relationship SP	software-based patient communication interface and collection of patient-related data	Alexa, iOS, Apple Health App, Microsoft Healthcare Bot, Amazon Marketplace
data processing & exchange SP	storage and standardization of patient data and API provision	Alexa, iOS, Apple Health App, Microsoft Azure API
clinical data processing SP	collection, storage, preparation, and processing of large and cross-sectional clinical or medical data sets for analytical purposes	Amazon Comprehend Medical, Facebook FAIR, Microsoft Azure
AI support SP	provision of specialized health-related AI systems, applications, and algorithms	Amazon Comprehend Medical, Facebook FAIR, Microsoft Healthcare Bot

## Construction of the platform-induced value network

**Fig. 5:**
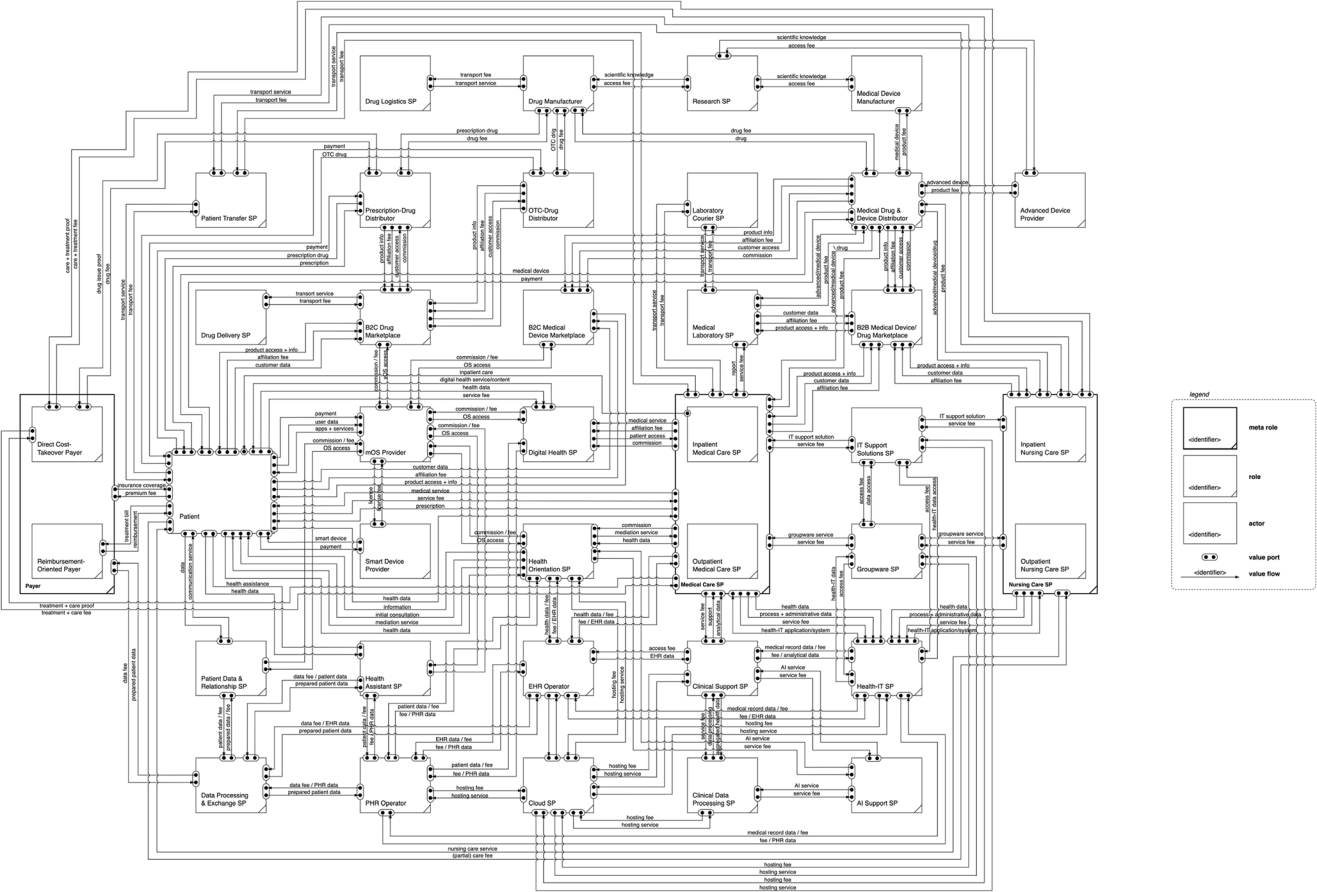
Platform-induced healthcare market value network

The resulting platform-induced roles need to be thoughtfully integrated into the healthcare market value network with special regard to their relationships with other roles. Therefore, we refer again to value flow analysis and the extended e3-value modeling method to develop the value network of the platform-induced healthcare market based on the defined roles and their value linkages. For instance, the mOS provider gives patients access to their platform (and thus, to various services from other providers). In return, a patient transfers data and payment fees. Table 10 exhibits exemplary value linkages within the value network of the platform-induced healthcare market. Through carefully connecting all these value linkages among the entities, we are able to construct the resulting role-based value network of the platform-induced healthcare market (see Fig. 5).

**Table 10:** Excerpt of defined value linkages in the conventional healthcare market value network

From role/actor/segment	Value linkage	To role/actor
patient	access/service fee; data	mOS provider
mOS provider	OS access and infrastructure; access to services and products	patient
mOS provider	access to patients and devices	digital health SP
digital health SP	access/commission fee	mOS provider

In the center, we find the patient who demands a healthcare service. The ubiquity of digital technologies in general and GAFAM platforms in particular enables new and more complex patient pathways. If digitally empowered (through mOS and smart devices), patients are given several opportunities which neutralize the necessity of contacting healthcare SP directly. New roles between this traditional relationship evolve and do not only provide patient-oriented ecosystems but also low-threshold orientation and access to information or consultation, mostly in exchange for data and usage fees. Conversely, healthcare SP (voluntarily or not) access these platforms from the opposite side to get in touch with patients or offer digital services themselves (e.g., telemedicine). From a B2B perspective, GAFAM platforms offer fee-based services for healthcare SP which facilitate the health-related and administrative business activities (e.g., clinical support SP, groupware provider, PHR operator). Consequently, the products and services of GAFAM platforms orbit patients and healthcare SP likewise, which breaks ground for a central and intermediary position in the value network and enables the successive connection to other value-creation segments in the network (e.g., drug and medical device market platforms). In combination with third-party developers and service or content providers, GAFAM platforms might pursue full-service approaches for patients (and for healthcare SP). GAFAM platforms also supply relevant infrastructures, ecosystems, and advanced services that lay the foundation for comprehensive and interconnected products and services (e.g., mOS provider, patient data aggregator, AI support SP).

GAFAM platforms are not limited to creating value in healthcare by enabling transaction, but also provide information and facilitate interaction among different stakeholders (both B2B and B2C). As the value network analysis illustrates, data might play a key part in the GAFAM-related actions in the healthcare value network, especially in terms of value collection, exchange, and analytics. On the one hand, this enables platforms to provide various digital and data-driven service types such as intermediation, content delivery, or communication. On the other hand, GAFAM platforms can support and facilitate physical service types along the entire healthcare process cycle and for all kinds of healthcare services from monitoring to therapy. Plus, GAFAM platforms exploit their existing customer relationships, networks, and ecosystems to interact with different user groups from different market segments. Besides, their technological capabilities allow for various kinds of customer and network interaction from automated chats to face-to-face video calls.

Both patients and healthcare SP conduct value exchange (data, money, services, products) with GAFAM platforms at different tie points. Hence, value streams rise in frequency and complexity, making data an important (or even decisive) competitive factor. The defined value exchange linkages are represented through the value network of the platform-induced healthcare market. With respect to our research aim, we employ this value network in the following sections to illustrate, analyze, and discuss the platform impact on value creation in healthcare.

## Analysis of GAFAM impact on healthcare value creation

### Classification of GAFAM healthcare services

To analyze the platform-induced impact on the healthcare market systematically, we first apply the morphological method. We conceptualize and typologize the business models of the examined platform healthcare services and the substantiated roles and value flows in the reference model. Thus, we derive appropriate characteristics for classifying platform healthcare service types and assign them to the three dimensions of digital transformation, i.e., value creation model (VCM), value proposition model (VPM), and customer interaction model (CIM) (Pousttchi 2017; Täuscher and Laudien 2018). While the VCM refers to a firm’s architecture and processes that enable and ensure the value generation, the VPM explains how the generated value can be offered and transformed into revenues and profits, including its revenue streams and sources. Complementarily, the CIM describes how the generated value is sustainably delivered to the customers, including the channels and segments (Abdelkafi and Täuscher 2016; Johnson et al. 2008; Pousttchi 2017).

**Fig. 6:**
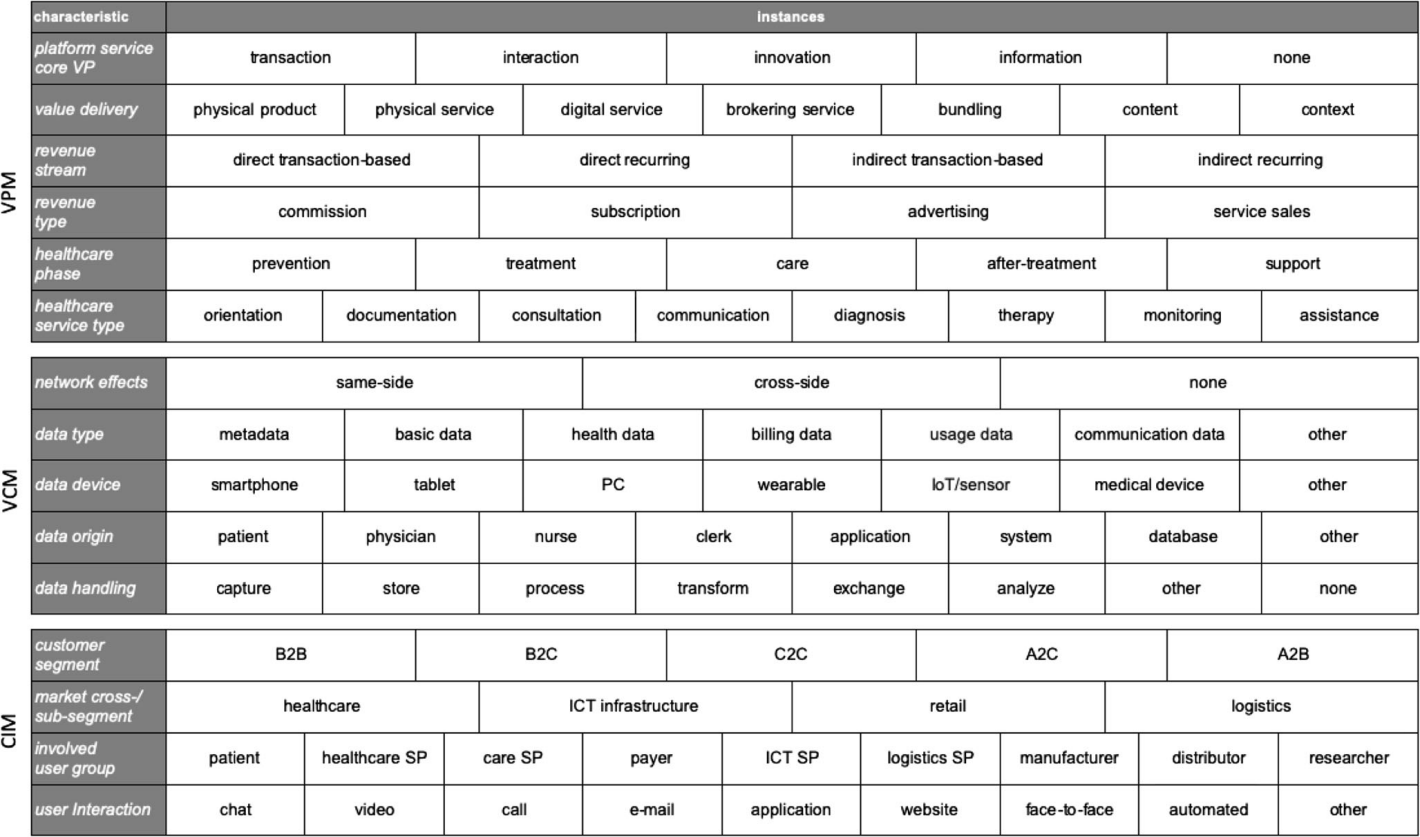
Classification of platform business models in healthcare

We first approach this structuring process deductively and draw on available literature. While Osterwalder (2004) and Pousttchi (2017) provide categorizing items of digital business models in general, Täuscher and Laudien (2018) propose distinct attributes of marketplace business models in particular, e.g., key activities, key value- proposition or key revenues. Since big digital platforms in healthcare are not limited to marketplaces, the derivation of further distinct characteristics requires an iterative process with an interplay of inductive and deductive elements. Thus, we grasp apparent features from the collected GAFAM activities in order to reflect, refine and substantiate those with available research on platform characteristics and business models (Eurich et al. 2011; Fehrer et al. 2018; Hein et al. 2020; Parker et al. 2016) as well as processes and market specifics in healthcare (Bergman et al. 2011) or relevant stakeholders (Donaldson and Preston 1995). This way, we derive and develop distinguishing characteristics and their range of possible instances of platform-induced roles in the healthcare market. The results are conceptualized to a morphological box and subdivided into the three dimensions of digital transformation (see Fig. 6).

#### Value proposition model

Regarding in the VPM, we can define the service’s *key value proposition*, i.e., either enabling transaction (e.g., B2C drug marketplace), facilitating interaction (e.g., digital health SP), fostering innovation (e.g., mOS provider), or providing information (e.g., health orientation SP). Upon this, we can determine the platform service’s graspable *value delivery*, which can be either physical or digital. Physical value deliveries imply tangible products (e.g., medical devices, smart devices, pharmaceutical drugs) or intangible professional services (e.g., health consultation), while digital ones imply distinct digital services (e.g., cloud services), brokering services (e.g., app store), bundling services (e.g., drug sales including delivery) or accessible content (i.e., standardized information like training videos) and context (i.e., personalized information like PHR). Although GAFAM platforms conventionally focus on digital value deliveries, our analyses reveal how they noticeably shift and connect to the physical world in healthcare with their (medical) devices and services, or hospitals and health insurance companies.

Further differentiation applies to the revenue model. At this, *revenue streams* can be direct or indirect (depending on who pays) and transaction-based (e.g., one-time fee) or recurring (e.g., monthly fee). Remarkably, GAFAM platforms receive their revenues both directly from the users (i.e., patients or hospitals) and indirectly from advertisers or insurances. *Revenue types* depend on the product or service and can be instantiated into commission, subscription, advertisi,ng and sales. GAFAM platforms basically generate revenues from brokering services (commission) and analyzing customer preferences (advertising), advanced healthcare services might be subject to subscription, especially in the context of B2B. Moreover, each platform service can directly or indirectly be assigned to a *healthcare phase* (i.e., prevention, treatment, care, after-treatment, support) and a *healthcare service type* (e.g., documentation, diagnosis, monitoring).

#### Value creation model

In terms of the VCM, platforms generate value through leveraging *network effects*, same-side or cross-side. For instance, the mOS provider creates both network effect types by connecting patients both among themselves (same-side) and to digital health or healthcare SP (cross-side) via access to portals for mobile applications. Moreover, big digital platforms create value by exploiting the power of (patient) data, which vary in their type, device, origin, and handling.

Regarding the *data type*, GAFAM platforms collect and process meta-data by their very nature, including information on what applications consumers use when and where, or exchange with whom. Additionally, GAFAM platforms can gather basic, communication, usage, and billing data from consumers: Facebook with its social network and WhatsApp, Apple with iOS and Google with Android (including messenger, call, and mobile-payment services), Amazon with Alexa and its marketplace, Microsoft with Windows. Stepping into the healthcare market and capturing new roles (e.g., health assistant SP), GAFAM platforms broaden and diversify their data pools to lay the foundations for more holistic big data approaches. To collect data (and process it on-premise), the platforms rely on a distributed infrastructure of *device types*, which they either provide themselves (e.g., Apple iPhones) or in cooperation (e.g., Google Android and Samsung). Generally, such devices include smartphones, tablets, or wearables. However, they increasingly provide medical devices or extract data from the same. The data can be either entered into the devices by humans (i.e., patients or staff) or generated automatically by the inherent applications. Thus, the *data origin* varies. Depending on the role in the value network (and therefore the required set of activities), GAFAM platform services pursue different purposes of *data handling*, e.g., the focus might be capturing and processing data (mOS provider) or storing, transforming, and exchanging data (patient data aggregator and data processing & exchange SP). Consequently, value creation from GAFAM platforms is highly reliant on the exploitation of data.

#### Customer interaction model

With respect to the CIM, we can specify the platform service’s *customer segment* (among business, consumers, and administration) and *market segment*, which can be close to the patient (healthcare) or at the periphery (i.e., ICT, retail and logistics). While GAFAM platforms have mainly arisen through their technological and data-processing capabilities (Google, Apple, Facebook, Microsoft) or logistic optimization (Amazon), our network analyses exhibit that they successively delve into directly healthcare-related activities from different corners in the value network. Plus, they diffuse all customer segments likewise, and therefore increasingly connect B2C and B2C. Irrespective of who pays for a service, different *user groups* might be involved and serviced. For instance, a telemedicine service might be charged to the payer but used by patients and physicians. Hence, several stakeholder types have to be taken into consideration when offering a health-related service. *User interaction* might also take place through different channels, depending on the technical capabilities and contextual requirements. For instance, a telemedical diagnosis service might require a high-resolution video stream between patient and physician, while a symptom checker might work chat-based. GAFAM platforms have the technological capacities to develop and offer such channels and services successfully, also with regard to the required minimum user base.

Altogether, GAFAM platforms offer a broad range and diversity of healthcare-related products and services to both patients and healthcare SP. Plus, these services do not necessarily constitute platform-specific value offerings. However, all GAFAM platforms and their products and services either rely or zero in on the collection, processing, analysis, or exchange of data.

### Analysis and discussion on GAFAM impact in healthcare

The value network analysis of the platform-induced healthcare market reveals that GAFAM platforms affect value creation in healthcare multifacetedly since they do not simply extend the conventional value network from various tie points but also raise its complexity through modularization. Furthermore, our morphological analysis of the GAFAM platform services in healthcare demonstrates how GAFAM platforms exploit their technological and data-processing capabilities as well as their large and diversified user bases to offer a broad spectrum of service types directly or indirectly related to healthcare. On that basis, this section aims to analyze and conceptualize the digital-platform impact on the healthcare market.

**Fig. 7:**
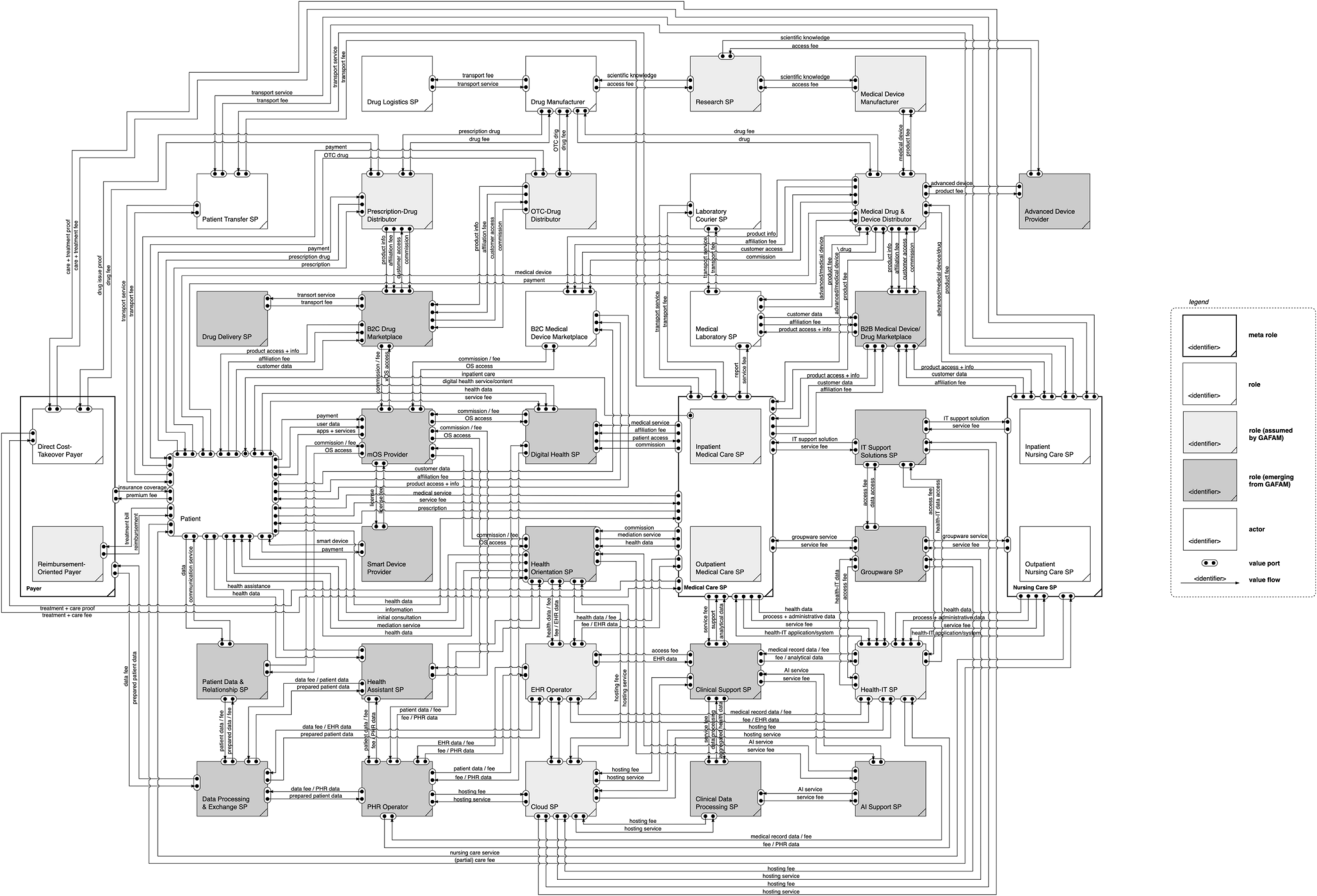
GAFAM roles in the platform-induced healthcare market

In order to understand the platform impact on value creation in the healthcare market in greater detail, we employ the platform-induced healthcare market value network as a starting point and tool for our further analysis (Fig. 5). We now ascribe GAFAM business activities to their respective roles in the value network to illustrate 1) which new roles have emerged from GAFAM, and 2) which conventional roles have been occupied by GAFAM (as depicted in Fig. 7). We find that GAFAM platforms mainly create new roles in the core and the ICT infrastructural periphery of the value network to connect or support conventional roles. Unsurprisingly, their core services represent digital gatekeepers to the patient. This primarily applies to the mOS provider (i.e., mainly Apple and Google), which interlinks all digitally-enabled services from the SP to the patient device, and thus combines much of the gathered data. What is more, other GAFAM services above the OS also tend to platformize and digitalize the B2C healthcare market by either bundling data from services and devices (e.g., Apple Health App), or intermediate services and products to the patient (e.g., Google Assistant, Amazon Marketplace).

However, GAFAM platforms also provide digital solutions for the healthcare SP, and thus compete with incumbent health IT companies. Either way, GAFAM platforms come from their core industries (i.e., ICT, data, retail, logistics) and rely on their core assets (i.e., technological and data-processing capabilities, consumer relationships). This way, GAFAM platforms might throw a bridge between B2B and B2C in the healthcare market.

More surprisingly, GAFAM platforms even capture conventional roles beyond their actual core industries and assets. For instance, Amazon may occupy the role of a payer (based on the project insights of Haven), and Apple and Amazon platforms accomplish the tasks of medical and care SP with their clinics, hospitals, and telemedicine services (AC Wellness and Care). This way, they penetrate the healthcare market both horizontally and vertically by extending their original service scope. Consequently, GAFAM platforms seize large parts of the healthcare market value network, break up existing relationship structures, and modularize value creation through intermediation or supportive functions. Altogether, GAFAM platforms affect the healthcare market in many ways, which requires a comprehensive organization of our findings. Thus, we develop a conceptual framework that structures and contextualizes the *facilitators* of the increasing platform occurrence in healthcare, enabled *GAFAM-specific healthcare activities*, and resulting *GAFAM-induced effects* on the healthcare market.

#### Facilitators

First, we propose six co-dependent facilitators which promote the prevalence of big digital platforms in healthcare according to our literature and value network analysis. (1) GAFAM platforms can exploit their *existing strong customer relationships* to offer health-related services to a broad user group: Apple provides a harmonious and comprehensive ecosystem of OS and customer devices (iOS, macOS), Google maintains the most widespread non-proprietary mOS and predominant information portals (e.g., Search, Maps), Facebook owns the most popular social network and messenger service (WhatsApp), Amazon is a global market leader for online shopping (Marketplace) and cloud services (AWS), and Microsoft runs the most successful office and collaboration software with integrated cloud services (Microsoft 365). On the one hand, people are used to the services and trust these actors. On the other hand, the success relies on homing and switching barriers. The manifold tie points to the patient in the platform-induced value network support this assumption. What is more, such strong customer relationships might spill over to B2B, if employees adopt their consumer behavior to work.

(2) GAFAM platforms have an enormous global ubiquity, pervasion, and *cross-sectional market power*, which goes back to the winner-takes-all phenomenon in platform markets. They can offer their ICT and data-based solutions (e.g., cloud services) to different markets, and thus achieve economies of scale by leveraging further network effects for their user groups. This implies a great bargaining power over other stakeholders (i.e., competitors, market participants, legal authorities, potential partners) to assert their claims and interests, or in terms of undertaking M&A transactions. This inference is indicated by the disparate business market segments (e.g., logistics, ICT) that GAFAM platforms target in the value network, bringing a multitude of different services to the patient and healthcare SP.

(3) The literature and morphological analyses exhibit that GAFAM platforms have tremendous *technological* and (4) *data collection and processing capabilities* which they can realize in healthcare. Especially, Google and Apple can draw on the most variable and voluminous data sets of content and metadata from their users. However, Amazon (billing data), Facebook (communication data), and Microsoft (online usage data) can almost hold their pace since services get increasingly extended (e.g., Alexa) and entangled (e.g., cookies). The GAFAM companies have proven to be able to manage such great amounts of data successfully, and for other market participants, it might be challenging to match up.

(5) As stated in the background section, the healthcare market is quite fragmented. The health IT sector has some big incumbent players (e.g., Cerner, AllScripts), but thousands of specialized IT SP. Apart from that, we find a plethora of hospitals, clinics, physicians, payers, or specialized manufacturers, and service providers (i.e., complementing participants) in the healthcare market. Thus, the (digital transforming) healthcare market provides an atomistic competition on the demand and supply side, which in turn is a fruitful basis for platform activities. Our value network analysis reveals that GAFAM platforms have several tie points to the healthcare market, and they will find several new roles in the healthcare market to control (i.e., occupy or interconnect), which might result in new value creation structures. While the GAFAM platforms mainly focus on their core assets (i.e., capabilities and market power), they also put efforts into research and development to discover future health technologies timely.

(6) The literature review revealed that digital technologies and data will play a vital role in the future of healthcare. The value network analysis illustrates that data turns out to be a key element of the platforms’ value exchange as GAFAM platforms mainly do not create value themselves but either intermediate or support others’ value-creating activities with their data processing and technological platform capabilities. On the one hand, the combination and quality of these capabilities are hardly imitable. On the other hand, their capabilities are sufficiently generic to cover, support, or connect a plethora of digitally enabled services in the healthcare market. In effect, GAFAM platforms might play a relevant part as data becomes of increased economic relevance.

Recent discussions on tracking and tracing COVID-19 infection chains have unfolded how those six facilitators intertwine and pertain in practice. Governments have realized soon that they would need (6) digitally gathered data to trace contact chains. However, it was virtually impossible to develop a technological solution without (1 + 2) Apple’s or Google’s ubiquitous mobile ecosystems and (3 + 4) their data collection and processing capabilities. Thus, (2) their market power allowed them to decide about opening the Bluetooth API, making entire states dependent on their goodwill. Google and Apple promptly decided on a tracking concept, (5) while other publicly financed collaboration initiatives were still debating about whether or not centralizing the storage of data (e.g., PEPP-PT). Hence, GAFAM platforms enter a promising field in which they can successively play off their strengths despite the regulatory issues and seemingly established market structures.

#### Activities

Second, we identified and propose three main co-dependent activities that sufficiently explain why GAFAM platforms might be successful in healthcare. (1) Typical of platforms, they smoothly edge into existing value-exchange relationships and make transactions more convenient or efficient. As the platform-induced value network analysis indicates, this particularly applies to the traditional patient-physician relationship. For instance, platforms can help patients to find physicians or hospitals and facilitate digitally enabled healthcare services (e.g., digital health SP). However, other supporting and transactional relationships are also subject to new intermediation services (e.g., B2B and B2C marketplaces for drug and medical devices, health data exchange between healthcare SP and digital health SP). Thus, it is remarkable to what extent GAFAM platforms place themselves in-between to break existing structures and *untie conventional relationships*.

(2) The value network analysis of the platform-induced healthcare market indicates that GAFAM platforms pounce on the healthcare market from several corners with direct (e.g., consultation, information, treatment, decision-support) *and* indirect healthcare-related business activities (e.g., administration, infrastructure, commerce) to underpin their market dominance. Thus, they both screw in-between existing relationship structures (e.g., patient and healthcare SP) and assume supportive or infrastructural roles in the periphery (e.g., Cloud SP, Research SP, AI Support SP, Logistics SP). Therefore, GAFAM platforms are not limited to a specific market segment (e.g., health, ICT, retail) but cover a broad spectrum of segments to offer their products and services. This way, GAFAM platforms can ensure full-service by expanding horizontally (regardless of whether they offer the actually demanded service themselves or not).

(3) What is more, GAFAM platforms are no longer limited to create value by enabling transaction (in terms of intermediation) among market participants, but also by providing products and content themselves (e.g., advanced device provider) that go beyond their core services. For instance, Apple provides the Health App as an upscale (platform) service based on iOS and corresponding devices. At the same time, it offers Health Records to clinics, and development kits to researchers and developers. Thus, GAFAM platforms can provide full-service approaches by extending their service range and expanding vertically through ecosystems of products, services, and content.

Altogether, GAFAM platforms pursue a product- and market-oriented growth strategy: They bring their existing products to the healthcare market (e.g., Microsoft 365), develop new services and products for healthcare providers and patients (e.g., Amazon Comprehend Medical or Basic Care), or develop entirely new value deliveries for future market segments (e.g., Facebook CRTL-kit or Calico).

#### Effects

Third, we uncovered and propose three main effects that result from the digital-platform impact on the healthcare market. (1) The platform-induced value network analysis shows that GAFAM platforms are about to control customer (or patient) relationships in several ways: As a first contact point for patients, they can govern the relationship between patients and healthcare service providers. Thus, they exploit their existing customer relations to untie conventional relationships in healthcare. Further governed relationships include and are not limited to: patient to patient, healthcare SP to healthcare SP, patient to manufacturers, healthcare SP to manufacturers, patient to digital health providers. Patients might hardly be able to avoid digital platforms when requesting digital (or digitally enabled) healthcare services. Likewise, other market participants might be dependent on digital platforms to reach the patient or access digital healthcare-related services. As a result, GAFAM platforms *monopolize the patient and customer ownership* and gather all possible data.

(2) What is more, GAFAM platforms might foster the *digitalization and platformization of healthcare services*. On the one hand, this implies the consumer-driven digital transformation in healthcare. GAFAM platforms do either provide digital health services themselves (e.g., Google Assistant and Fit) or platform access to remote digitalized services (e.g., Amazon Care, PillPack), as the value network analysis reveals. On the other hand, GAFAM platforms provide digital services and infrastructures for healthcare SP, and thus promote the digitalization and platformization from B2B. For instance, Microsoft provides an entire ecosystem of services that support clinical management (ehCOS EHR), IT management (Azure), collaboration (Windows, Teams, Surface), customer interaction (Healthcare Bot), and research and education (HoloLens).

(3) Our value network analysis demonstrates that GAFAM platforms also raise the complexity of healthcare value creation by fragmenting and modularizing the value creation processes. The increase of new roles within the platform-induced value network supports this assumption strongly. Thus, patients have a multitude of possibilities to access healthcare services and pass through digitally-enabled pathways. For one thing, this favors the development of new services. For another thing, conventional services can be offered and conducted in several ways. Likewise, healthcare SP is reliant on a proper ecosystem of ICT infrastructures, systems, and services to upheave healthcare into the digital era.

**Fig. 8:**
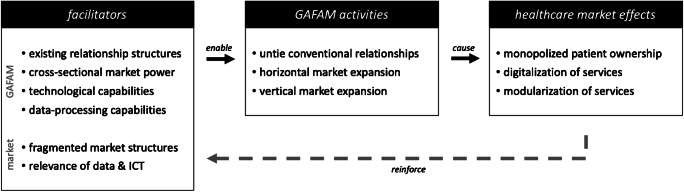
Healthcare GAFAM-impact framework

Altogether, we propose that big digital platforms might exploit their capacities and assets as facilitators to expand their business activities manifold to healthcare, and leverage powerful effects to the market in terms of customer interaction, digitalization, and service modularization (see Fig. 8). What is more, these effects might even reinforce the facilitating factors through the exploitation of further network and homing effects. In particular, the monopolized consumer ownership supports the existing relationship structures and cross-sectional power of GAFAM platforms, if both patients and healthcare SP might receive everything from one source or touchpoint. The gathered health-related data and new requirements in healthcare might strengthen the data-processing and technological capabilities of GAFAM platforms, which in turn might further increase the relevance of data and health IT. Plus, new platform market segments with modular services might further affect the market fragmentation with a multitude of specialized product service providers.

Our findings substantiate available research on digital platforms and digital transformation in healthcare and extend the current knowledge by connecting the tie points of both research avenues from a holistic standpoint. For one thing, our findings fit in seamlessly with existing observations and theories on the transformative impact of the platform economy on entire established markets and industries in view of their infrastructure and intermediation capabilities (Bakos and Katsamakas 2008; Parker et al. 2016; Hein et al. 2020). Particularly, GAFAM platforms have developed such capabilities that they can poly-directionally bring in to healthcare as our value network analysis illustrates. Hence, they might exploit their control over the consumer interface to tap new sources of data and revenue, and explore new opportunities for growth (which are rendered by current developments in healthcare).

For another thing, in reflection of the existent research on digital platforms in healthcare, our results confirm that GAFAM platforms could both solve and arouse different problems of the ongoing transformation in this sector. As central infrastructure providers and efficient intermediators, they help addressing the identified problems concerning interoperability, networking, and inter-sectoral communication (Estrin and Sim 2010; Fürstenau and Auschra 2016; Aanestad et al. 2019). Thus, GAFAM platforms could help innovating and coordinating the highly fragmented healthcare market (Fürstenau et al. 2019) but, on the contrary, they modularize the markets and attain control over critical digital resources (e.g., mOS, app stores, device interfaces) which might impede collaborative innovation. In so doing, they co-develop new market segments and value streams (Hermes et al. 2020), but largely either at the expense of the healthcare providers or the patients (in monetary or privacy terms). The patients foster these developments themselves through the increasing usage of digital-health apps, which supports recent literature in this research domain (e.g., Gu and Hong 2019; Huang et al. 2018). However, as research on digital health has mainly concentrated on the potentials of digital platforms in advancing collaboration and healthcare per se, we extend this knowledge by providing some contrasting insights into the potential threats to value creation in the healthcare market through the poly-lateral integration of the GAFAM platforms.

What is more, our findings support the state of research and practice on digital transformation in healthcare and the part of big digital platforms in it. Digital technologies will diffuse the healthcare market, and GAFAM platforms possess the economic and technological power to help address infrastructural issues in terms of standards, interoperability, collaboration, orchestration, or datafication. The fragmented healthcare market might be particularly receptive to such platform technologies and services, and GAFAM platforms are increasingly shifting to B2B markets. What is more, digital transformation in healthcare is patient-driven to a great extent and our value network analysis undermines how GAFAM platforms control access to patient-related digital services and data. Consequently, our results and inferences help to explain how GAFAM platforms might transform value creation in the healthcare market by not only introducing new roles in the value network but also by assuming formerly existing ones. This supports the assumption that GAFAM platforms probably will play an important part in the (digital) transformation of the healthcare market.

## Conclusion

The starting point of our consideration was the notion of increasing digital-platform activities in healthcare, which already undergoes a massive (digital) transformation. But despite the vast extent of platform literature and the risen research interest in the field of digital health, available research provides little insight on how big digital platforms transform value creation in healthcare. Against this background, our paper aimed to explore the economic impact of the five most powerful and valuable platforms, namely Google, Apple, Facebook, Amazon, and Microsoft, on the healthcare market. For that purpose, we relied on value network analyses of the healthcare market to explore how GAFAM services and products induce new value-creating roles and mechanisms in healthcare. Hereupon, we examined the GAFAM-impact on healthcare by scrutinizing the facilitators, activities, and effects, which we condensed into a conceptual framework.

Our findings suggest that GAFAM platforms are about to affect value creation in healthcare in various ways as they edge into existing structures by not only enabling transaction and interaction but also providing content and products themselves. At this, they target several market segments from direct healthcare and digital health to peripheral activities such as logistics, retail, and R&D, which results in ubiquitous platform involvement in the entire healthcare market. Platforms control customer relationships and network structures from all sides – and right from the midst of the patient-physician relationship. The appearance of big digital platforms ascribes data a key part in future value propositions and value linkages. All combined, the digital platforms simultaneously centralize the customer relationship and modularize value creation in healthcare by introducing new roles to the value network. To leverage these effects, GAFAM platforms highly benefit from their cross-sectional market power, pervasiveness, and existing customer relationships and network structures in their core industries, which helps them to successively infuse the healthcare market. Additionally, the platforms reveal enormous data collection capacities (in both scope and variety) they can combine with high-end data processing and analytics, which is advantageous in this highly fragmented healthcare market.

Taken as a whole, GAFAM platforms might affect value creation in healthcare severely and bear the potential to transform at least parts of the market. Patients are facing a multitude of new services and channels to manage their health condition, and new ways of patient interaction might stimulate the occurrence of new business models. At the same time, healthcare service providers increasingly become dependent on platforms in terms of their value creation, value proposition, and patient interaction. Consequently, shifts in the balance of powers within the value network and changing customer behaviors will lead to new constellations of customer relationship.

As a contribution to research, we connect and extend current knowledge on digital platforms and digital transformation in healthcare by providing a new holistic perspective on how big digital platforms might transform value-creation structures in this market. Particularly, we explain how GAFAM platforms contribute to this transformation by exploiting their cross-sectional and technological capacities. Thus, we provide a new standpoint when assessing the potentials and threats of digital platforms in approaching the challenges of digital health. Furthermore, we add new insights into the transformative impact of big digital platforms on conventional, service-oriented markets and confirm research findings from other industries. Our reference models might be employed for further analyses to either explore new business models for and beyond platforms in healthcare or to examine the platform-induced socio-economic impact on traditional healthcare structures (e.g., patient-physician relationship). Thus, our contribution adds another puzzle piece in understanding the complex coherences among economy, society, and technology of platform-induced shifts in entire markets. As a contribution to practice, we might sensitize managers and policymakers in healthcare about the increasing and ubiquitous influence of platforms on their domain, and emphasize the importance of collaboration and shared standards. Hence, practitioners could employ the reference models to assess platform-related threats and opportunities or redesign their business models and tap new (data-driven) revenue sources.

Our study has several limitations. First, the scope of our study is limited to GAFAM platforms. Thus, our findings and inferences might not be fully applicable to other digital platforms, be it smaller platforms in the Western economy or big digital platforms of the Asian economic area (e.g., Tencent, Alibaba). Second, our qualitative research design, including the value network development and analysis, is reliant upon the subjective selection, coding, and interpretation of the authors. In awareness of this limitation, our findings build upon reliable research sources and practical evidence. Plus, we validated our findings with both industry and academic experts. Third, we have not investigated how incumbent players, both healthcare and technology providers, should strategically respond to the digital transformation in general and the emergence of GAFAM platforms in particular. Forth and most importantly, we acknowledge the willful neglect of the regulatory body for reasons of generalizability, which entails barriers for both GAFAM platforms and incumbent players in healthcare.

We see three avenues for future research. First, it could further elaborate on the potential implications of *GAFAM platforms in healthcare*. In particular, we should investigate the role of data and personalized services to formulate platform strategies for incumbent players in healthcare. At this, we could rely on available findings or long-term studies from other industries. Likewise, we should factor in regulation and liability in healthcare, which might provide insights for platform regulation in other industries. Second, future research could explore the impact of digital platforms *beyond GAFAM in healthcare* since the emergence of digital technologies might leverage further dynamics within the conventional value creation structures. This applies to a more technological standpoint on how digital platforms might effectively contribute to the digital transformation in healthcare. Third and more holistically, future research could explore the socio-economic impact of *GAFAM platforms beyond healthcare*. After all, it is remarkable how GAFAM platforms have infused entire industries, and how they might honeycomb the healthcare market almost unnoticed and from different tie points. Possibly, big digital platforms might turn from market participants to market owners. This again gives reason to expect equally severe economic shifts in value creation structures of other conventional markets, which could be substantiated with econometric models.

## Supplementary Information

ESM 1 (DOCX 20581 kb)

ESM 2 (PDF 243 kb)

ESM 3 (PDF 243 kb)

ESM 4 (PDF 243 kb)

ESM 5 (PDF 244 kb)

ESM 6 (PDF 243 kb)

## Abbreviations

API: Application Programming Interface
AI: Artificial Intelligence
B2A: Business-to-Administration (Market Segment)
B2B: Business-to-Business (Market Segment)
B2C: Business-to-Consumer (Market Segment)
BI: Business Informatics
CIM: Customer Interaction Model
CIO: Chief Information Officer
COVID-19: Coronavirus Disease SARS-CoV-2
DSR: Design Science Research
EHR: Electronic Health Record
FHIR: Fast Healthcare Interoperability Resources
GAFAM: Google, Apple, Facebook, Amazon, Microsoft
GI: German Informatics Society
HCI: Human-Computer Interaction
HIPAA: Health Insurance Portability and Accountability Act (in the U.S.)
HIS: Health Information System
HIT: Health Information Technologies
ICT: Information and Communications Technologies
IoT: Internet of Things
IS: Information Systems
IT: Information Technologies
M&A: Mergers & Acquisitions
mHealth: Mobile Health
OS: Operating System
OTC: Over-the-Counter
PHR: Personal Health Record
PHTI: Public Health Tech Initiative (from the Consumer Technology Association)
SP: Service Provider
VCM: Value Creation Model
VPM: Value Proposition Model
WHO: World Health Organization

## References

[CR1] Aanestad, M., Vassilakopoulou, P., & Øvrelid, E. (2019). Collaborative innovation in healthcare: Boundary resources for peripheral actors. *Proceedings of the* *40*^*th*^*International Conference on Information Systems (ICIS)*, Munich, Germany. https://aisel.aisnet.org/icis2019/is_health/is_health/24.

[CR2] Abdelkafi, N., & Täuscher, K. (2016). Business models for sustainability from a system dynamics perspective. *Organization & Environment, 29*(1), 74–96. 10.1177/1086026615592930.

[CR3] Abdelkafi, N., Raasch, C., Roth, A., & Srinivasan, R. (2019). Multi-sided platforms. *Electronic Markets, 29*(4), 553–559. 10.1007/s12525-019-00385-4.

[CR4] Agarwal, R., Gao, G., DesRoches, C., & Jha, A. K. (2010). Research commentary—The digital transformation of healthcare: Current status and the road ahead. *Information Systems Research, 21*(4), 796–809. 10.1287/isre.1100.0327.

[CR5] Alt, R., & Zimmermann, H.-D. (2019). Electronic markets on platform competition. *Electronic Markets, 29*(2), 143–149. 10.1007/s12525-019-00353-y.

[CR6] Armstrong, M. (2006). Competition in two-sided markets. *The Rand Journal of Economics, 37*(3), 668–691. 10.1111/j.1756-2171.2006.tb00037.x.

[CR7] Asadullah, A., Faik, I., & Kankanhalli, A. (2018). Digital platforms: A review and future directions. *PACIS 2018 Proceedings. *https://aisel.aisnet.org/pacis2018/248

[CR8] Bakos, Y., & Katsamakas, E. (2008). Design and ownership of two-sided networks: Implications for internet platforms. *Journal of Management Information Systems, 25*(2), 171–202. 10.2753/MIS0742-1222250208

[CR9] Bandyopadhyay, S., Ozdemir, Z., & Barron, J. (2012). The future of personal Health Records in the Presence of misaligned incentives. *Communications of the Association for Information Systems*, *31*. 10.17705/1CAIS.03107.

[CR10] Barbier-Feraud, I., Bossy Malafosse, J., Bouexel, P, Commaille-Chapus, C., Gimalac, A., et al. (2016). *Big data and prevention from prediction to demonstration*. White Paper of the Healthcare Data Institute*.* https://healthcaredatainstitute.com/wp-content/uploads/2017/01/hdi-bigdata-prevention-2016-final.pdf. Accessed 30 Oct 2020.

[CR11] Basole, R. C., & Rouse, W. B. (2008). Complexity of service value networks: Conceptualization and empirical investigation. *IBM Systems Journal, 47*(1), 53–70. 10.1147/sj.471.0053.

[CR12] Baumöl, U., Hollebeek, L., & Jung, R. (2016). Dynamics of customer interaction on social media platforms. *Electronic Markets, 26*(3), 199–202. 10.1007/s12525-016-0227-0.

[CR13] Becker, J., & Schütte, R. (2004). *Handelsinformationssysteme (Information Systems in Retail)*. Landsberg (Germany): Verlag moderne Industrie.

[CR14] Bender, B. (2020). The impact of integration on application success and customer satisfaction in Mobile device platforms. *Business & Information Systems Engineering, 62*(6), 515–533. 10.1007/s12599-020-00629-0.

[CR15] Bergman, B., Neuhauser, D., & Provost, L. (2011). Five main processes in healthcare: A citizen perspective. *BMJ Quality & Safety, 20*(Suppl 1), i41–i42. 10.1136/bmjqs.2010.046409. 10.1136/bmjqs.2010.046409 PMC3066700 21450769

[CR16] Bernus, P. (1999). *GERAM: **Generalised Enterprise reference architecture and methodology. IFIP–IFAC Task Force on Architectures for Enterprise Integration* (Technical Report). 10.13140/RG.2.2.35937.33120.

[CR17] Bharadwaj, A., El Sawy, O. A., Pavlou, P. A., & Venkatraman, N. (2013). Digital business strategy: Toward a next generation of insights. *MIS Quarterly, 37*(2), 471–482. 10.25300/MISQ/2013/37:2.3.

[CR18] Blechman, E. A., Raich, P., Raghupathi, W., & Blass, S. (2012). Strategic value of an unbound, interoperable PHR platform for rights–managed care coordination. *Communications of the Association for Information Systems*, *30*. 10.17705/1CAIS.03006.

[CR19] Bowman, C., & Ambrosini, V. (2000). Value creation versus value capture: Towards a coherent definition of value in strategy. *British Journal of Management, 11*(1), 1–15. 10.1111/1467-8551.00147.

[CR20] Burton-Jones, A., Akhlaghpour, S., Ayre, S., Barde, P., Staib, A., & Sullivan, C. (2020). Changing the conversation on evaluating digital transformation in healthcare: Insights from an institutional analysis. *Information and Organization, 30*(1), 100255. 10.1016/j.infoandorg.2019.100255.

[CR21] CB Insights (2018). *Google strategy teardown*. CB Insights Core Intelligence Reports. https://www.cbinsights.com/reports/CB-Insights_Core-Intelligence_Google-Strategy-Teardown.pdf?utm_campaign=google-teardown_2018-08&utm_medium=email&_hsenc=p2ANqtz-_OTRV02h_cyo1PL_Z1cvD107d3rRzqTcPocIW2DUB5y2XiiffrzLa0Wh4WEkN0bhuI9g_lBmOGifTXgLBXeTEh77Pk_g&_hsmi=65261034&utm_content=65261034&utm_source=hs_automation&hsCtaTracking=a70526ae-d3c0-40c1-92ef-bfd4b319395c%7C969db417-49f4-42d8-9ffb-bc3b5757865c. Accessed 15 Oct 2020.

[CR22] CDPH – California Department of Public Health (2020). *Difference between hospital and clinic*. CDPH Glossary*.* https://www.cdph.ca.gov/Programs/CID/ORH/Pages/Difference-between-Hospital-and-Clinic.aspx. Accessed 20 March 2020.

[CR23] Chan, R. (2020). *Amazon’s cloud generated over $10 billion in net quarterly sales for the first time ever — up 33% from a year ago* . Business Insider. https://www.businessinsider.com/amazon-earnings-aws-amazon-web-services-10-billion-quarterly-revenue-2020-4?r=DE&IR=T#:~:text=Amazon%20Web%20Services%2C%20the%20retailer’s,recorded%20revenue%20of%20%249.95%20billion. Accessed 17 10 2020.

[CR24] Chen, A. (2019). *As tech companies move into health care, here’s what to watch in 2019*. The Verge. https://www.theverge.com/2019/1/3/18166673/technology-health-care-amazon-apple-uber-alphabet-google-verily. Accessed 22 Oct 2020.

[CR25] Christensen, C. M., & Rosenbloom, R. S. (1995). Explaining the attacker’s advantage: Technological paradigms, organizational dynamics, and the value network. *Research Policy, 24*(2), 233–257. 10.1016/0048-7333(93)00764-K.

[CR26] Cohen, A. B., Dorsey, E. R., Mathews, S. C., Bates, D. W., & Safavi, K. (2020). A digital health industry cohort across the health continuum. *npj Digital Medicine, 3*, 68. 10.1038/s41746-020-0276-9. 32411829 10.1038/s41746-020-0276-9 PMC7217869

[CR27] Coldewey, D., Korosec, K., Heater, B., & Matney, L. (2020). *Four perspectives on Apple's new service bundle*. TechCrunch. https://techcrunch.com/2020/09/16/four-perspectives-on-apples-new-service-bundle/. Accessed 17 Oct 2020.

[CR28] Correa, D. (2020). *U.S. Healthcare IT Market Worth $149.17 Billion by 2025*. Bloomberg PR newswire. https://www.bloomberg.com/press-releases/2020-02-25/u-s-healthcare-it-market-worth-149-17-billion-by-2025-amr. Accessed 17 Oct 2020.

[CR29] De Benedictis, A., Lettieri, E., & Masella, et al. (2019). WhatsApp in hospital? An empirical investigation of individual and organizational determinants to use. *PLoS One, 14*(1), e0209873. 10.1371/journal.pone.0209873. 30633754 10.1371/journal.pone.0209873 PMC6329505

[CR30] de Reuver, M., Sørensen, C., & Basole, R. C. (2018). The digital platform: A research agenda. *Journal of Information Technology, 33*(2), 124–135. 10.1057/s41265-016-0033-3.

[CR31] Dehling, T., & Sunyaev, A. (2014). Secure provision of patient-centered health information technology services in public networks—Leveraging security and privacy features provided by the German nationwide health information technology infrastructure. *Electronic Markets, 24*(2), 89–99. 10.1007/s12525-013-0150-6.

[CR32] Dimitrov, D. V. (2016). Medical internet of things and big data in healthcare. *Healthcare Informatics Research, 22*(3), 156–163. 10.4258/hir.2016.22.3.156. 27525156 10.4258/hir.2016.22.3.156 PMC4981575

[CR33] Dolata, U. (2017). Apple, Amazon, Google, Facebook, Microsoft: Market concentration – competition – innovation strategies. *SOI Discussion Paper* (2017-01). http://hdl.handle.net/10419/152249.

[CR34] Donaldson, T., & Preston, L. E. (1995). The stakeholder theory of the corporation: Concepts, evidence, and implications. *The Academy of Management Review, 20*(1), 65–91. 10.2307/258887.

[CR35] Drees. J. (2019). *Google receives more than 1 billion health questions every day*. Becker’s Hospital Review. https://www.beckershospitalreview.com/healthcare-information-technology/google-receives-more-than-1-billion-health-questions-every-day.html. Accessed 15 Oct 2020.

[CR36] Durst, C., Viol, J., & Wickramasinghe, N. (2013). Online social networks, social capital and Health-related behaviors: A state-of-the-art analysis. *Communications of the Association for Information Systems*, *32*. 10.17705/1CAIS.03205.

[CR37] Dyrda, L. (2020). *The Most dangerous Health IT trends: Insights from 8 execs*. Becker’s Hospital Review. https://www.beckershospitalreview.com/healthcare-information-technology/the-most-dangerous-health-it-trends-insights-from-8-execs.html. Accessed 15 Oct 2020.

[CR38] Eisenmann, T., Parker, G., & van Alstyne, M. W. (2006). *Strategies for Two-Sided Markets. Harvard Business Review*, 12.

[CR39] Enderle, R. (2019). *How Microsoft failed with windows 10 Mobile*. ComputerWorld*.* https://www.computerworld.com/article/3336057/how-microsoft-failed-with-windows-10-mobile.html. Accessed 18 Oct 2020.

[CR40] Estrin, D., & Sim, I. (2010). Open mHealth architecture: An engine for Health care innovation. *Science, 330*(6005), 759–760. 10.1126/science.1196187. 21051617 10.1126/science.1196187

[CR41] Eurich, M., Giessmann, A., Mettler, T., & Stanoevska-Slabeva, K. (2011). Revenue streams of cloud-based platforms: Current state and future directions. *AMCIS 2011 Proceedings.* https://aisel.aisnet.org/amcis2011_submissions/302.

[CR42] Evans, P. C., & Gawer, A. (2016). The rise of the platform Enterprise: A global survey. In: *The emerging platform economy series, 1*. The Center for Global Enterprise (CGE), New York (NY).

[CR43] Evans, D. S., Hagiu, A., & Schmalensee, R. (2006). *Invisible engines: How software platforms drive innovation and transform industries*. Cambridge (MA): MIT Press.

[CR44] Fagherazzi, G., Goetzinger, C., Rashid, M. A., Aguayo, G. A., & Huiart, L. (2020). Digital Health strategies to fight COVID-19 worldwide: Challenges, recommendations, and a call for papers. *Journal of Medical Internet Research, 22*(6), e19284. 10.2196/19284. 32501804 10.2196/19284 PMC7298971

[CR45] Fehrer, J. A., Woratschek, H., & Brodie, R. J. (2018). A systemic logic for platform business models. *Journal of Service Management, 29*(4), 546–568. 10.1108/JOSM-02-2017-0036.

[CR46] Fettke, P., & Loos, P. (2004). Referenzmodellierungsforschung (Reference Modeling Research). *Wirtschaftsinformatik, 46*(5), 331–340. 10.1007/BF03250947.

[CR47] Fettke, P., & Loos, P. (Eds.). (2007). *Reference modeling for business systems analysis*. Hershey (PA) and London (UK): Idea Group Pub.

[CR48] Fürstenau, D., & Auschra, C. (2016). Open digital platforms in Health care: Implementation and scaling strategies. *Proceedings of the 37*^*th*^*International Conference on Information Systems (ICIS)*, Dublin, Ireland.

[CR49] Fürstenau, D., Auschra, C., Klein, S., & Gersch, M. (2019). A process perspective on platform design and management: Evidence from a digital platform in health care. *Electronic Markets, 29*(4), 581–596. 10.1007/s12525-018-0323-4.

[CR50] Galloway, S. (2018). *The four – The hidden DNA of Amazon, Apple, Facebook, and Google*. New York (NY): Portfolio/Penguin.

[CR51] Gawer, A. (2014). Bridging differing perspectives on technological platforms: Toward an integrative framework. *Research Policy, 43*(7), 1239–1249. 10.1016/j.respol.2014.03.006.

[CR52] Gerke, S., Stern, A. D., & Minssen, T. (2020). Germany’s digital health reforms in the COVID-19 era: Lessons and opportunities for other countries. *npj Digital Medicine 3*, 94. 10.1038/s41746-020-0306-7. 10.1038/s41746-020-0306-7 PMC7351985 32685700

[CR53] Gibbs, S. (2015). *Google to put health information directly into search results*. The Guardian. https://www.theguardian.com/technology/2015/feb/10/google-health-information-directly-into-search-results. Accessed 3 May 2020.

[CR54] GoHealth. (2017). *Urgent Care vs. Emergency room: What’s the difference?* GoHealth Urgent Care. https://www.gohealthuc.com/UCvsER. Accessed 12 March 2020.

[CR55] Google Health (2020). *Our mission*. Google Health. https://health.google/. Accessed 7 Nov 2020.

[CR56] Gopal, G., Suter-Crazzolara, C., Toldo, L., & Eberhardt, W. (2019). Digital transformation in healthcare – Architectures of present and future information technologies. *Clinical Chemistry and Laboratory Medicine, 57*(3), 328–335. 10.1515/cclm-2018-0658. 30530878 10.1515/cclm-2018-0658

[CR57] Gordijn, J., Akkermans, H., & Van Vliet, H. (2000). What’s in an electronic business model? In R. Dieng & O. Corby (Eds.), *EKAW 2020: Knowledge engineering and knowledge management methods, models, and tools *(pp. 257–273). Springer. 10.1007/3-540-39967-4_19.

[CR58] Gregg, H. (2015). *50 things to know about epic, Cerner, MEDITECH, McKesson, athenahealth and other major EHR vendors*. Beckert’s Hospital Review. https://www.beckershospitalreview.com/healthcare-information-technology/50-things-to-know-about-epic-cerner-meditech-mckesson-athenahealth-and-other-major-ehr-vendors.html. Accessed 15 Oct 2020.

[CR59] Gregor, S. (2006). The nature of theory in information systems. *MIS Quarterly, 30*(3), 611–642. 10.2307/25148742.

[CR60] Gu, R., & Hong, Y. K. (2019). Addressing health misinformation dissemination on mobile social media.* Proceedings of the 40*^*th*^*International Conference on Information Systems (ICIS)*, Munich, Germany.

[CR61] Hagiu, A., & Wright, J. (2015). Multisided platforms. *HBS Working Paper.*

[CR62] Hein, A., Schreieck, M., Riasanow, T., Setzke, D. S., Wiesche, M., Böhm, M., & Krcmar, H. (2020). Digital platform ecosystems. *Electronic Markets, 30*(1), 87–98. 10.1007/s12525-019-00377-4.

[CR63] Hermes, S., Riasanow, T., Clemons, E. C., Böhm, M., & Krcmar, H. (2020): The digital transformation of the healthcare industry: Exploring the rise of emerging platform ecosystems and their influence on the role of patients. *Business Research,* (online first). 10.1007/s40685-020-00125-x.

[CR64] Hevner, A. R., March, S. T., Park, Y., & Ram, S. (2004). Design science in information systems research. *MIS Quarterly, 28*(1), 75–105. 10.2307/25148625.

[CR65] Hotie, F., & Gordijn, J. (2019). Value-based process model design. *Business & Information Systems Engineering, 61*(2), 163–180. 10.1007/s12599-017-0496-y.

[CR66] Househ, M., Borycki, E., & Kushniruk, A. (2014). Empowering patients through social media: The benefits and challenges. *Health Informatics Journal, 20*(1), 50–58. 10.1177/1460458213476969. 24550564 10.1177/1460458213476969

[CR67] Huang, W., Chen, H., & Kwon, J. (2018). The impact of Gamification design on the success of Health and fitness apps. *PACIS 2018 Proceedings.* https://aisel.aisnet.org/pacis2018/288.

[CR68] Jindal, P. (2019). *How Microsoft, Google, Apple, and Amazon are fueling FHIR*. Darena Solutions Blog*.* https://www.darenasolutions.com/blog/how-microsoft-google-apple-and-amazon-are-fueling-fhir. Accessed 30 Oct 2020.

[CR69] Johnson, M. W., Christensen, C. M., & Kagermann, H. (2008, December 1). Reinventing your business model. *Harvard Business Review*, December 2008. https://hbr.org/2008/12/reinventing-your-business-model

[CR70] Kagermann, H. (2015). Change through digitization—Value creation in the age of industry 4.0. In H. Albach, H. Meffert, A. Pinkwart, & R. Reichwald (Eds.), *Management of Permanent Change *(pp. 23–45). Springer Fachmedien. 10.1007/978-3-658-05014-6_2.

[CR71] Kaletsch, A., & Sunyaev, A. (2011). Privacy engineering: Personal Health Records in Cloud Computing Environments. *Proceedings of the 32*^*nd*^*International Conference on Information Systems (ICIS)*, Shanghai, China.

[CR72] Kawczynski, L., & Taisch, M. (2010). Health care provider value chain. In B. Vallespir & T. Alix (Eds.), *Advances in production management systems. New challenges, new approaches* (Vol. 338, pp. 611–618). Springer Berlin Heidelberg. 10.1007/978-3-642-16358-6_76.

[CR73] Kenney, M., & Zysman, J. (2016). The rise of the platform economy. *Issues in Science and Technology* (spring 2016), 61–69.

[CR74] Kimmell, J. (2019a).* The 5 ways Apple wants to transform health care*. Advisory Board. https://www.advisory.com/dailybriefing/2019/01/22/apple#:~:text=Apple’s%20future%20ambitions%20may%20be,improving%20medication%20adherence%20and%20diet. Accessed 15 Oct 2020

[CR75] Kimmell, J. (2019b). *The 5 latest health care moves by Amazon, Google, apple, and more*. Advisory Board*.* https://www.advisory.com/daily-briefing/2019/09/12/disruptor-watch. Aaccessed 15 Oct 2020.

[CR76] Kordzadeh, N., & Warren, J. (2017). Communicating personal Health information in virtual Health communities: An integration of privacy Calculus model and affective commitment. *Journal of the Association for Information Systems*, *18*(1), 45–81. 10.17705/1jais.00446

[CR77] Kuchler, H. (2020). *Can we ever trust Google with our health data?* Financial Times. https://www.ft.com/content/4ade8884-1b40-11ea-97df-cc63de1d73f4. Accessed 16 Oct 2020.

[CR78] Kuebel, H., Hanner, & Zarnekow, R. (2015). An expert view on the role of complementary assets for the adoption of smart home platforms. *PACIS 2015 Proceedings*. https://aisel.aisnet.org/pacis2015/56.

[CR79] Kuechler, W., & Vaishnavi, V. (2012). A framework for theory development in design science research: Multiple perspectives. *Journal of the Association for Information Systems, 13*(6), 395–423. 10.17705/1jais.00300.

[CR80] Kwon, H. E., Oh, W., & Kim, T. (2017). Platform structures, homing preferences, and Homophilous propensities in online social networks. *Journal of Management Information Systems, 34*(3), 768–802. 10.1080/07421222.2017.1373008.

[CR81] Lapāo, L. (2019). The future of healthcare: The impact of digitalization on healthcare services performance. In: A.P. Neto & M. B. Flynn (Eds.) *The internet and health in Brazil—challenges and trends* (pp. 435–449). Springer Nature. 10.1007/978-3-319-99289-1.

[CR82] Liu, X., Zhang, B., Susarlia, A., & Padman, R. (2020). Go to you tube and call me in the morning: Use of social Media for Chronic Conditions. *MIS Quarterly*, *44*(1), 257–283. 10.25300/MISQ/2020/15107.

[CR83] Lusch, R., & Vargo, S. L. (2006). Service-dominant logic: Reactions, reflections and refinements. *Marketing theory 6*(3), 281–288. 10.1177/1470593106066781.

[CR84] March, S. T., & Storey, V. C. (2008). Design science in the information systems discipline: An introduction to the special issue on design science research. *MIS Quarterly, 32*(4), 725–730. 10.2307/25148869.

[CR85] Matthews, K. (2020). *Google is building an EHR tool: What to know*. Electronic Health Reporter (MillerRupp). https://electronichealthreporter.com/google-is-building-an-ehr-tool-what-to-know/. Accessed 7 Nov 2020.

[CR86] Mayring, P. (2000). Qualitative content analysis. *Forum: Qualitative Social Research, 1*(2).

[CR87] Menvielle, L., Audrain-Pontevia, A.-F., & Menvielle, W. (2017). *The digitization of healthcare: New challenges and opportunities*. London (UK): Palgrave Macmillan. 10.1057/978-1-349-95173-4.

[CR88] Mol, J. M., Wijnberg, N. M., & Carroll, C. (2005). Value chain envy: Explaining new entry and vertical integration in popular music. *Journal of Management Studies, 42*(2), 251–276. 10.1111/j.1467-6486.2005.00496.x.

[CR89] Myers, M. D. (1997). Qualitative research in information systems. *MIS Quarterly, 21*(2), 241–242. 10.2307/249422.

[CR90] Myllärniemi, J., & Helander, N. (2012). Healthcare system as a value network. *World Review of Entrepreneurship, Management and Sustainable Development, 8*(2), 196–207. 10.1504/WREMSD.2012.046120.

[CR91] Myreteg, G. (2015). Cost-benefit evaluation of e-health services: Acceptance and value creation are interactive forces. *Health Systems, 4*(3), 204–211. 10.1057/hs.2015.10.

[CR92] Niemann, T., & Burghardt, T. (2016). German Healthcare System (Einführung in das deutsche Gesundheitswesen). *BodyLIFE*, *11/2016*.

[CR93] OASIS. (2020). *OASIS SOA reference models. SOA Reference Models (SOA-RM) TC (FAQ)**.* Oasis Open. https://www.oasis-open.org/committees/soa-rm/faq.php. Accessed 22 April 2020.

[CR94] Orlikowski, W. J., & Baroudi, J. J. (1991). Studying information Technology in Organizations: Research approaches and assumptions. *Information Systems Research, 2*(1), 1–28. 10.1287/isre.2.1.1

[CR95] Osterwalder, A. (2004). *The business model ontology—A proposition in a design science approach*. Switzerland: University of Lausanne.

[CR96] Otto, L., Harst, L. (2019). Investigating barriers for the implementation of telemedicine initiatives: A systematic review of reviews. *AMCIS 2019 Proceedings.*

[CR97] Pagani, M. (2013). Digital business strategy and value creation: Framing the dynamic cycle of control points. *MIS Quarterly, 37*(2), 617–632. 10.25300/MISQ/2013/37.2.13.

[CR98] Parker, G. G., & van Alstyne, M. W. (2005). Two-sided network effects: A theory of information product design. *Management Science, 51*(10), 1494–1504. 10.1287/mnsc.1050.0400.

[CR99] Parker, G. G., van Alstyne, M. W., & Choudary, S. P. (2016). *Platform revolution: How networked markets are transforming the economy and how to make them work for you*. W. W. Norton & Company, New York (NY).

[CR100] Pearl, R. (2019). *Why big tech companies Won’t solve Healthcare’s biggest challenges*. Forbes. https://www.forbes.com/sites/robertpearl/2019/12/16/big-tech/. Acessed 20 April.

[CR101] Peppard, J., & Rylander, A. (2006). From value chain to value network: Insights for Mobile operators. *European Management Journal, 24*(2–3), 128–141. 10.1016/j.emj.2006.03.003.

[CR102] Petit, N. (2016). Technology giants, the "Moligopoly" hypothesis and holistic competition: A primer. *Working Paper.* Availabe at SSRN. 10.2139/ssrn.2856502

[CR103] Pinna, R., Carrus, P. P., & Marras, F. (2015). The drug logistics process: An innovative experience. *The TQM Journal, 27*(2), 214–230. 10.1108/TQM-01-2015-0004.

[CR104] Pitta, D. A., & Laric, M. V. (2004). Value chains in health care. *Journal of Consumer Marketing, 21*(7), 451–464. 10.1108/07363760410568671.

[CR105] Pousttchi, K. (2008). A modeling approach and reference models for the analysis of mobile payment use cases. *Electronic Commerce Research and Applications, 7*(2), 182–201. 10.1016/j.elerap.2007.07.001.

[CR106] Pousttchi, K. (2017). Digitale transformation (digital transformation). In: Gronau, N., Becker, J., Kliewer, N., Leimeister, J.M., Overhage, S. (Eds.), Enzyklopädie der Wirtschaftsinformatik (encyclopedia of business informatics). http://www.enzyklopaedie-der-wirtschaftsinformatik.de/lexikon/technologien-methoden/Informatik%2D%2DGrundlagen/digitalisierung/digitale-transformation/digitale-transformation. Accessed 13 Feb 2020.

[CR107] Pousttchi, K., & Dehnert, M. (2018). Exploring the digitalization impact on consumer decision-making in retail banking. *Electronic Markets, 28*(3), 265–286. 10.1007/s12525-017-0283-0.

[CR108] Pousttchi, K., & Gleiss, A. (2019). Surrounded by middlemen - how multi-sided platforms change the insurance industry. *Electronic Markets, 29*(4), 609–629. 10.1007/s12525-019-00363-w.

[CR109] Pousttchi, K., & Hufenbach, Y. (2014). Engineering the value network of the customer interface and marketing in the data-rich retail environment. *International Journal of Electronic Commerce, 18*(4), 17–41. 10.2753/JEC1086-4415180401.

[CR110] Pousttchi, K., Schlieter, H., Gleiss, A. (Eds.) (2019). *Digitale Innovationen im Gesundheitsmarkt (Digital Innovation in Healthcare)*. GITO, Berlin (Germany).

[CR111] Raghupathi, W., & Raghupathi, V. (2014). Big data analytics in healthcare: Promise and potential. *Health Information Science and Systems, 2*(3). 10.1186/2047-2501-2-3. 10.1186/2047-2501-2-3 PMC4341817 25825667

[CR112] Raghupathi, W., & Tan, J. (2008). Information systems and healthcare XXX: Charting a strategic path for Health information technology. *Communications of the Association for Information Systems*, *23*. 10.17705/1CAIS.02328

[CR113] Ritchey, T. (2013). *General Morphological Analysis*: A general method for non-quantified modeling. *Swedish Morphological Society*. http://www.swemorph.com/ma.html

[CR114] Rochet, J.-C., & Tirole, J. (2003). Platform competition in two-sided markets. *Journal of the European Economic Association, 1*(4), 990–1029. 10.1162/154247603322493212.

[CR115] Safavi, K., & Kalis, B. (2020). *Digital health technology vision 2020*. Accenture White Paper*.* https://www.accenture.com/_acnmedia/PDF-130/Accenture-Health-Tech-Vision-2020.pdf#zoom=40. Accessed 26 Oct 2020.

[CR116] Schaarschmidt, M., Ivens, S., & Homscheid, D. (2017). Dr. miller or Dr. smith? Patients’ intentions to make appointments on physician rating platforms. *Proceedings of the 38*^*th*^ *International Conference on Information Systems (ICIS)*, Seoul, South Korea.

[CR117] Schlichter, B. R., Svejvig, P., & Andersen, P. E. R. (2014). Value creation from public healthcare IS. In: B. Bergvall-Kåreborn & P. A. Nielsen (Eds.), *Creating Value for All Through IT* (Vol. 429, pp. 1–15). Springer, Berlin, Heidelberg (Germany). 10.1007/978-3-662-43459-8_1.

[CR118] Schneider, S., & Sunyaev, A. (2015). CloudLive: A life cycle framework for cloud services. *Electronic Markets, 25*(4), 299–311. 10.1007/s12525-015-0205-y.

[CR119] Semuels, A. (2020). *Many companies won't survive the pandemic. Amazon Will Emerge Stronger Than Ever*. TIME. https://time.com/5870826/amazon-coronavirus-jeff-bezos-congress/. Accessed 18 Oct 2020.

[CR120] Shapiro, C., & Varian, H. R. (1998). *Information rules: A strategic guide to the network economy*. Boston (MA): Harvard Business Press.

[CR121] Sriram, S., Manchanda, P., Bravo, M. E., Chu, J., Ma, L., Song, M., Shriver, S., & Subramanian, U. (2015). Platforms: A multiplicity of research opportunities. *Marketing Letters, 26*(2), 141–152. 10.1007/s11002-014-9314-1.

[CR122] StatCounter (2020a). *Search engine market share worldwide*. https://gs.statcounter.com/search-engine-market-share. Accessed 29 2020.

[CR123] StatCounter (2020b). *Mobile operating system market share worldwide*. https://gs.statcounter.com/os-market-share/mobile/worldwide. Accessed 29 Oct 2020.

[CR124] Sutherland, W., & Jarrahi, M. H. (2018). The sharing economy and digital platforms: A review and research agenda. *International Journal of Information Management, 43*, 328–341. 10.1016/j.ijinfomgt.2018.07.004.

[CR125] Tan, B., Pan, S., Lu, X., & Huang, L. (2015). The role of IS capabilities in the development of multi-sided platforms: The digital ecosystem strategy of Alibaba.com. *Journal of the Association for Information Systems*, *16*(4), 248–280. 10.17705/1jais.00393.

[CR126] Täuscher, K., & Laudien, S. M. (2018). Understanding platform business models: A mixed methods study of marketplaces. *European Management Journal, 36*(3), 319–329. 10.1016/j.emj.2017.06.005.

[CR127] Tiwana, A., Konsynski, B., & Bush, A. A. (2010). Research commentary—Platform evolution: Coevolution of platform architecture, governance, and environmental dynamics. *Information Systems Research, 21*(4), 675–687. 10.1287/isre.1100.0323.

[CR128] Vaishnavi, V., Kuechler, B, & Petter, S. (2019). *Design Science Research in Information Systems* (created in 2004 and updated until 2015 by Vaishnavi, V. and Kuechler, W.); last updated (by Vaishnavi, V. and Petter, S.), . http://www.desrist.org/design-research-in-information-systems/. Accessed 30 June 2019.

[CR129] van der Aalst, W., Hinz, O., & Weinhardt, C. (2019). Big digital platforms: Growth, impact, and challenges. *Business & Information Systems Engineering, 61*(6), 645–648. 10.1007/s12599-019-00618-y.

[CR130] Vesselkov, A., Hämmäinen, H., & Töyli, J. (2019). Design and governance of mHealth data sharing. *Communications of the Association for Information Systems*, 299–321. 10.17705/1CAIS.04518

[CR131] Vincent, J. (2020). *Without Apple and Google, the UK’s contact-tracing app is in trouble*. The Verge*.* https://www.theverge.com/2020/5/5/21248288/uk-covid-19-contact-tracing-app-bluetooth-restrictions-apple-google. Accessed 18 Oct 2020.

[CR132] Vogel, D., Viehland, D., Wickramasinghe, N., & Mula, J. M. (2013). Mobile health. *Electronic Markets, 23*(1), 3–4. 10.1007/s12525-013-0121-y.

[CR133] vom Brocke, J. (2007). Design principles for reference modeling: Reusing information models by means of aggregation, specialisation, instantiation, and analogy. In P. Fettke & P. Loos (Eds.), *Reference modeling for business systems analysis* (pp. 47–76). Hershey (PA) and London (UK): Idea Group Pub. 10.4018/978-1-59904-054-7.ch003.

[CR134] Walters, D., & Jones, P. (2001). Value and value chains in healthcare: A quality management perspective. *The TQM Magazine, 13*(5), 319–335. 10.1108/EUM0000000005858.

[CR135] Weissinger, R. (2014). Economic benefits of standards. *PIK – Praxis Der Informationsverarbeitung und Kommunikation, 37*(3). 10.1515/pik-2014-0015.

[CR136] WHO – World Health Organization (2019).* Global Spending on Health: A World in Transition*. *WHO Global Report 2019*. https://www.who.int/health_financing/documents/health-expenditure-report-2019.pdf?ua=1. Accessed 31 Oct 2020.

[CR137] Wickramasinghe, N., & Kirn, S. (2013). E-Health and the future of healthcare information systems. *Business & Information Systems Engineering, 5*(1), 1–2. 10.1007/s12599-012-0245-1.

[CR138] Wilde, T., & Hess, T. (2007). Forschungsmethoden der Wirtschaftsinformatik. *Wirtschaftsinformatik*, 49(4), 280–287. 10.1007/s11576-007-0064-z.

[CR139] Willing, C., Brandt, T., & Neumann, D. (2017). Electronic mobility market platforms – A review of the current state and applications of business analytics. *Electronic Markets, 27*(3), 267–282. 10.1007/s12525-017-0257-2.

[CR140] Yin, R. K. (2009). *Case study research: Design and methods* (4th ed.). Thousand Oaks (CA): Sage Publications. 10.33524/cjar.v14i1.73.

[CR141] Zenooz, A. M., & Fox, J. (2019). How new Health care platforms will improve patient care. *Harvard Business Review*. https://hbr.org/2019/10/how-new-health-care-platforms-will-improve-patient-care. Accessed 12 Feb 2020.

[CR142] Zhang, X., Wu, Y., Valacich, J. S., Jenkins, J. L., & Li, K. (2019). How online patient–physician interaction influences service satisfaction. *Proceedings of the 40*^*th*^*International Conference on Information Systems (ICIS)*, Munich, Germany.

[CR143] Zwicky, F. (1966). *Entdecken, Erfinden, Forschen im Morphologischen Weltbild (the morphological method to discover, create, and research the world)* (2^nd^ ed.). Droemer-Knaur, Munich (Germany).

