# Supplementary material for: An apple a day – how the platform economy impacts value creation in the healthcare market
Source: Electron Mark. 2021 Apr 14;31(4):849–76. doi: 10.1007/s12525-021-00467-2 (PMC8043778; doi:10.1007/s12525-021-00467-2)

# Appendix

## Roles derived from GAFAM activities in healthcare

## Google


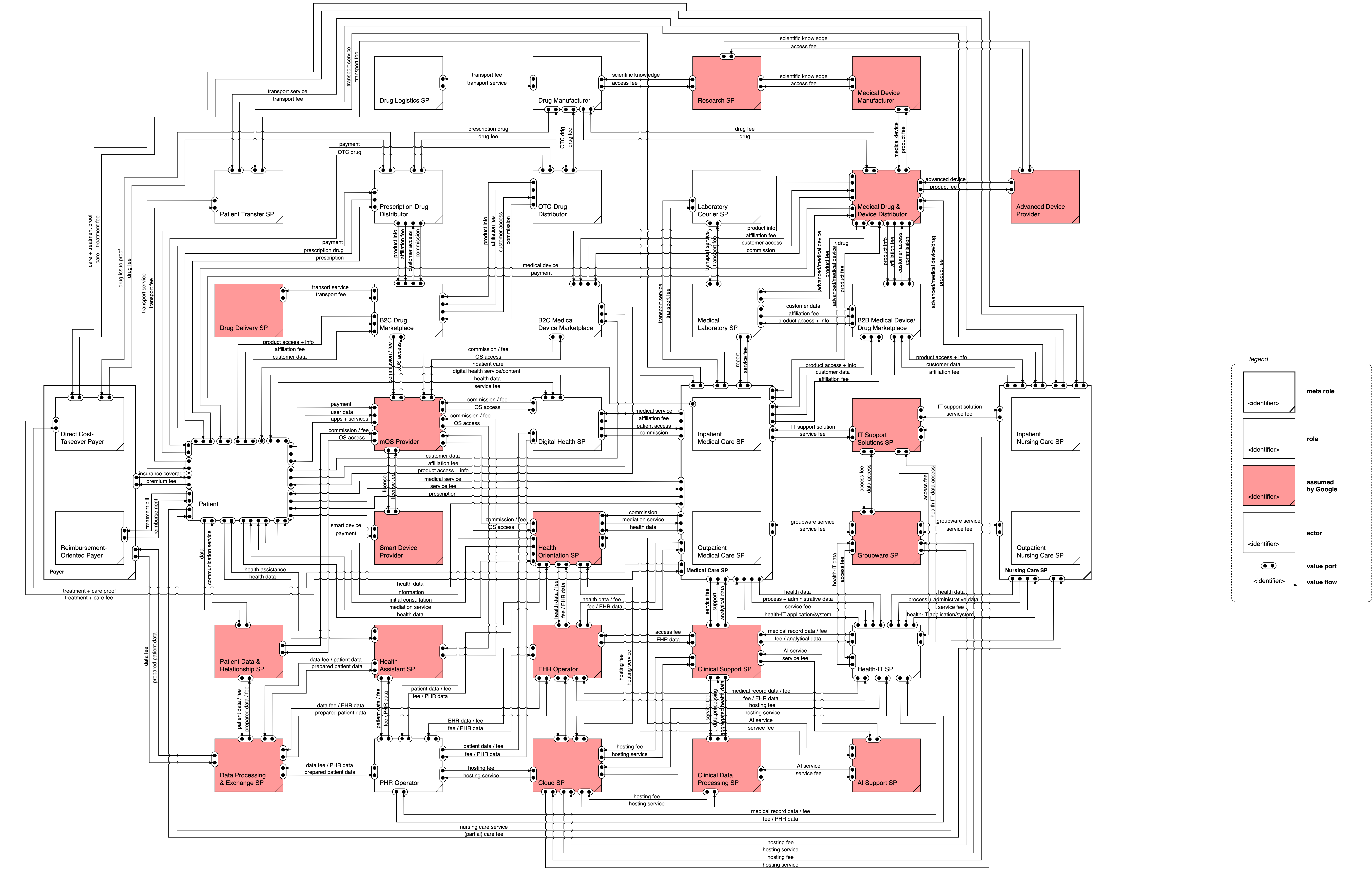


## Apple


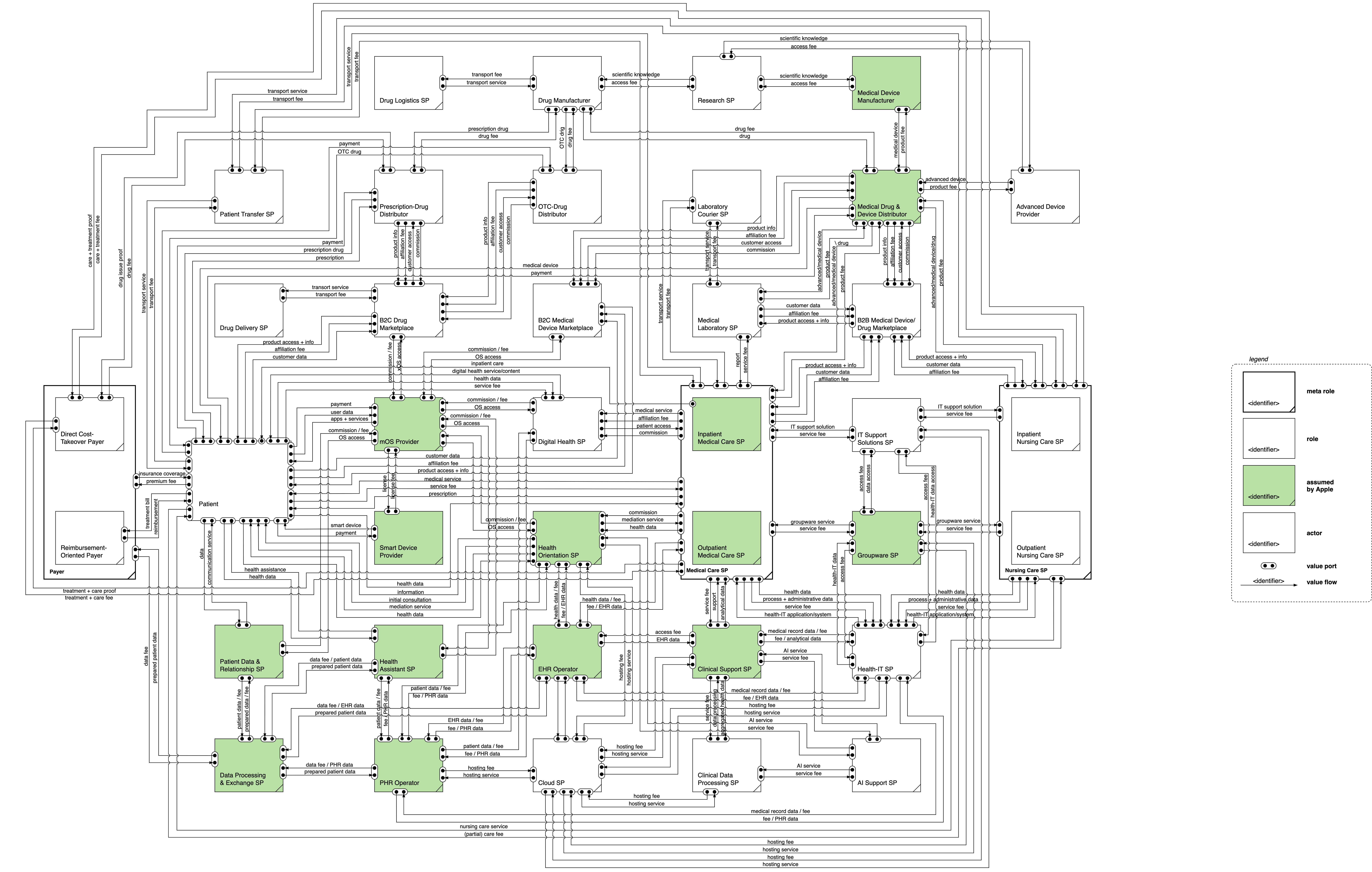


## Facebook


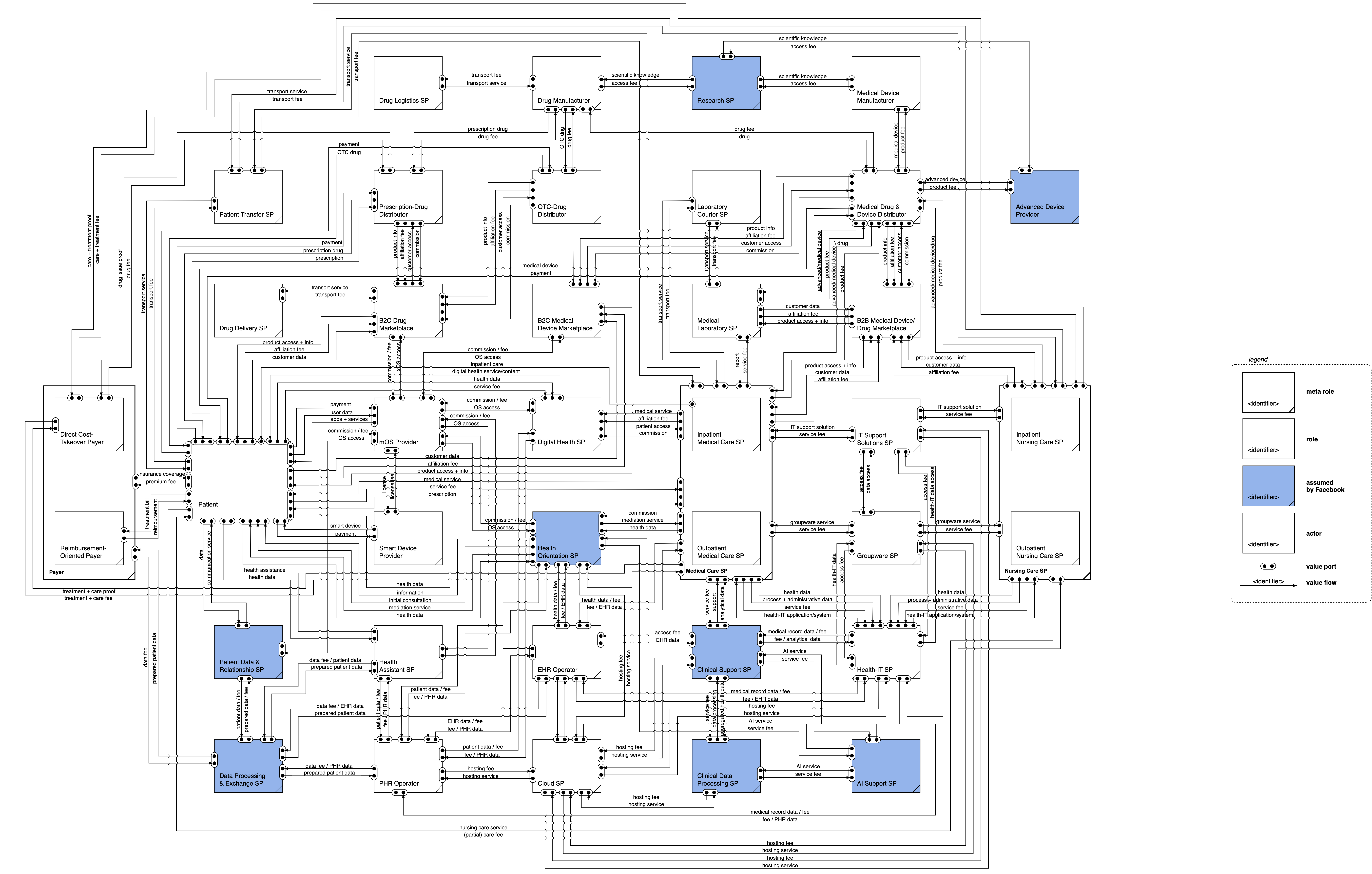


## Amazon


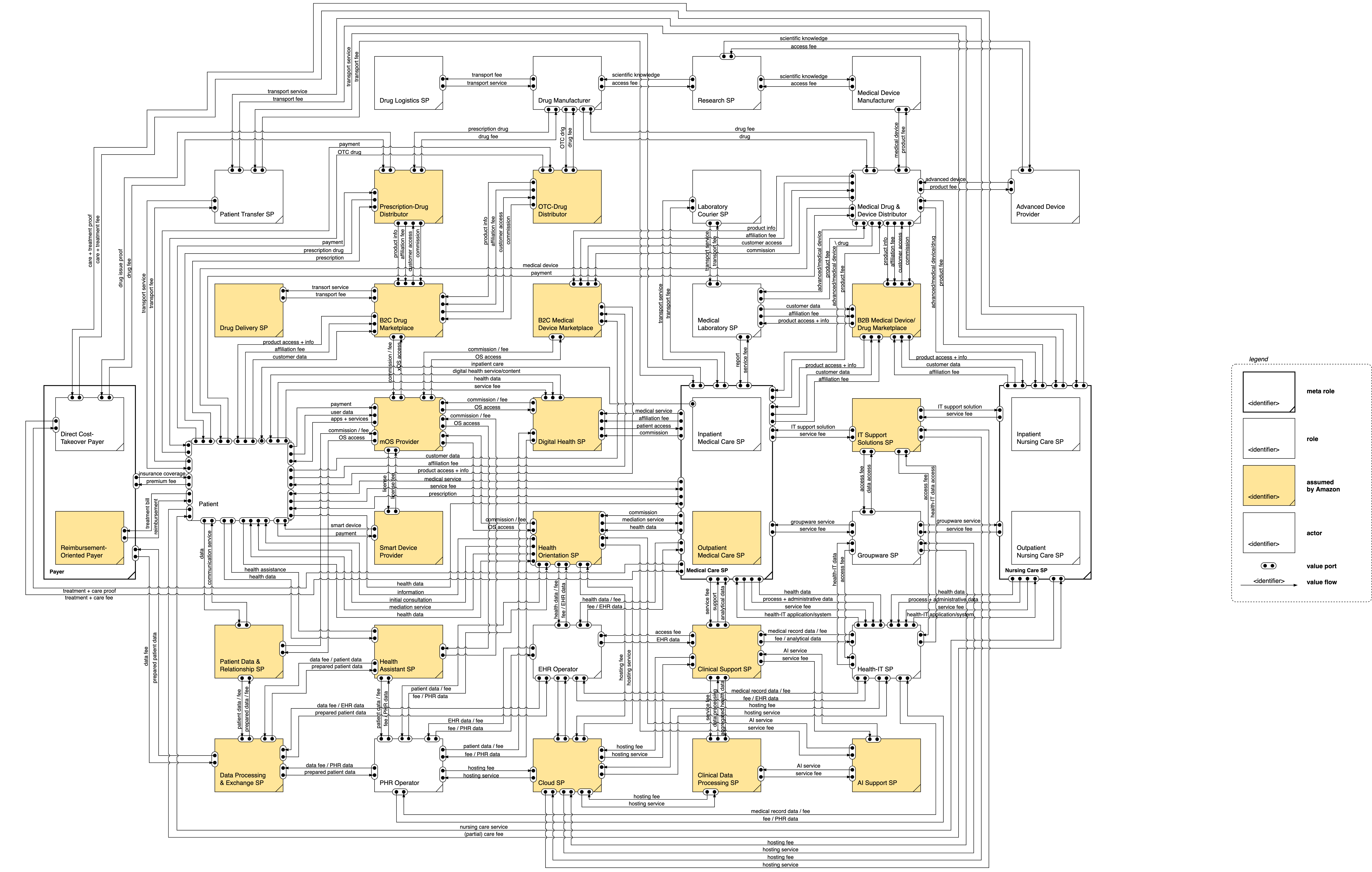


## Microsoft


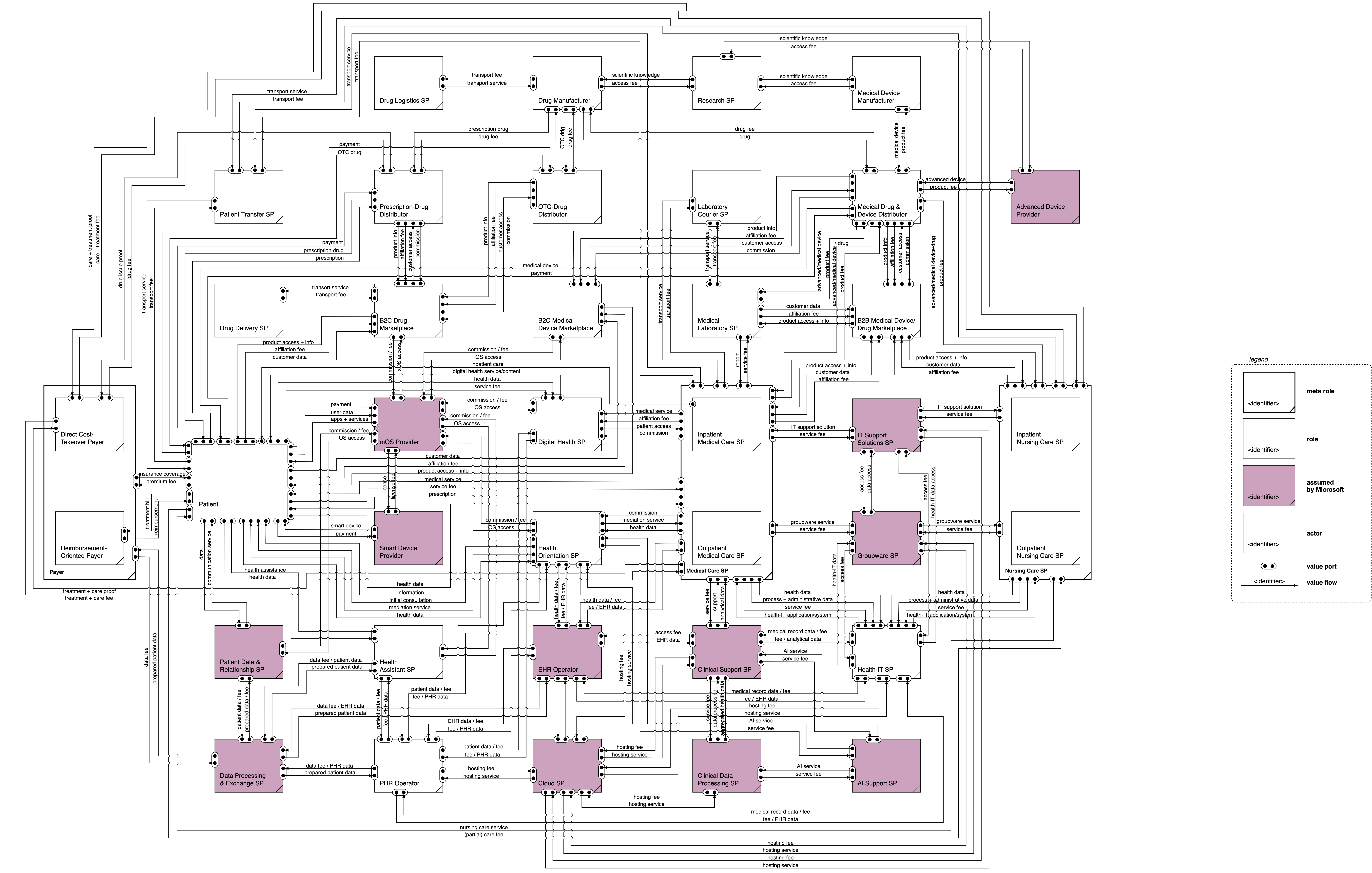

Supplement: Supplementary file 1 — (DOCX 20581 kb) [file 12525_2021_467_MOESM1_ESM.docx]
