# Supplementary figures and images for: An apple a day – how the platform economy impacts value creation in the healthcare market
Source: Electron Mark. 2021 Apr 14;31(4):849–76. doi: 10.1007/s12525-021-00467-2 (PMC8043778; doi:10.1007/s12525-021-00467-2)

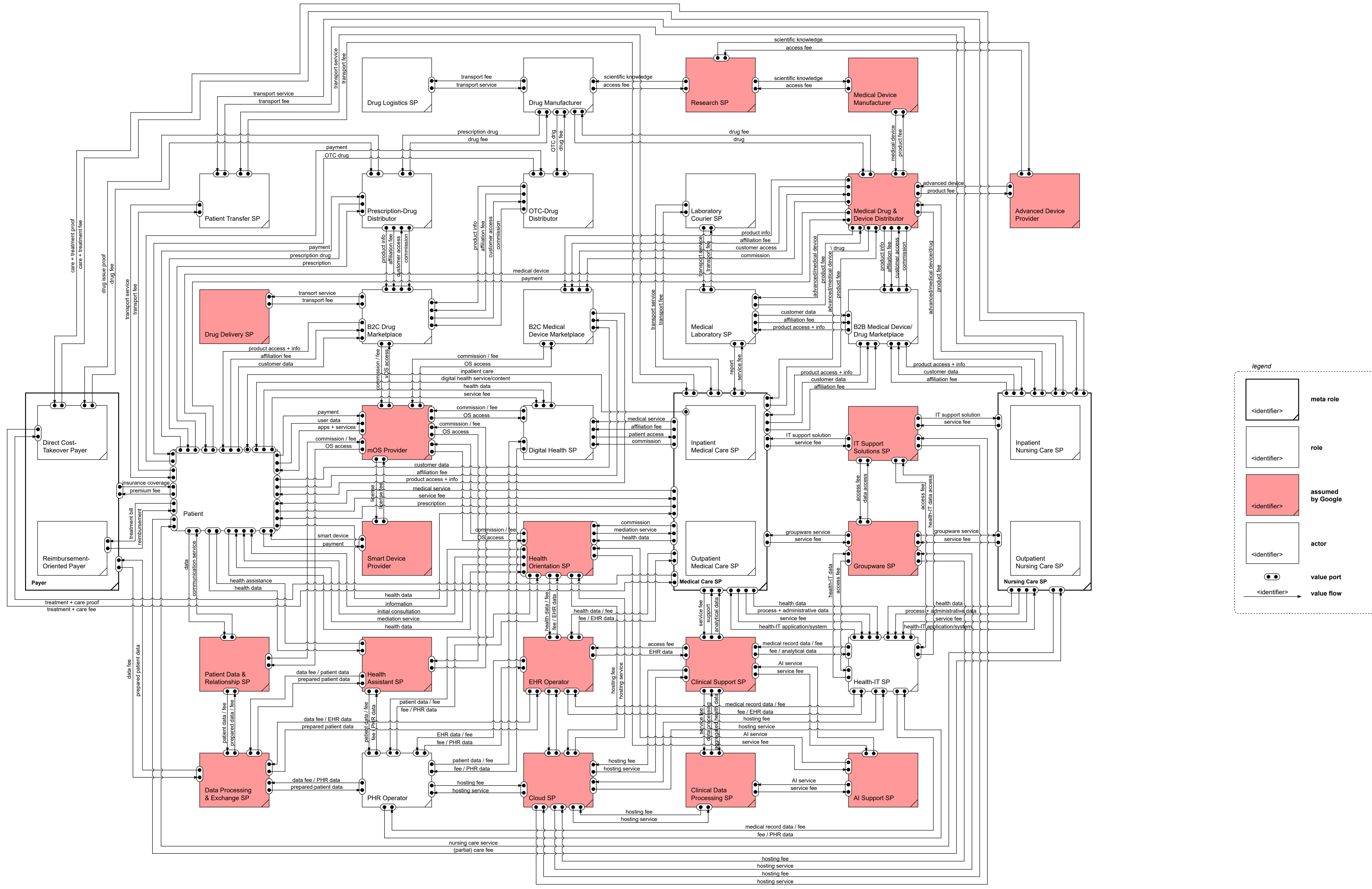

Supplement: Supplementary file 2 — (PDF 243 kb) [file 12525_2021_467_MOESM2_ESM.pdf]

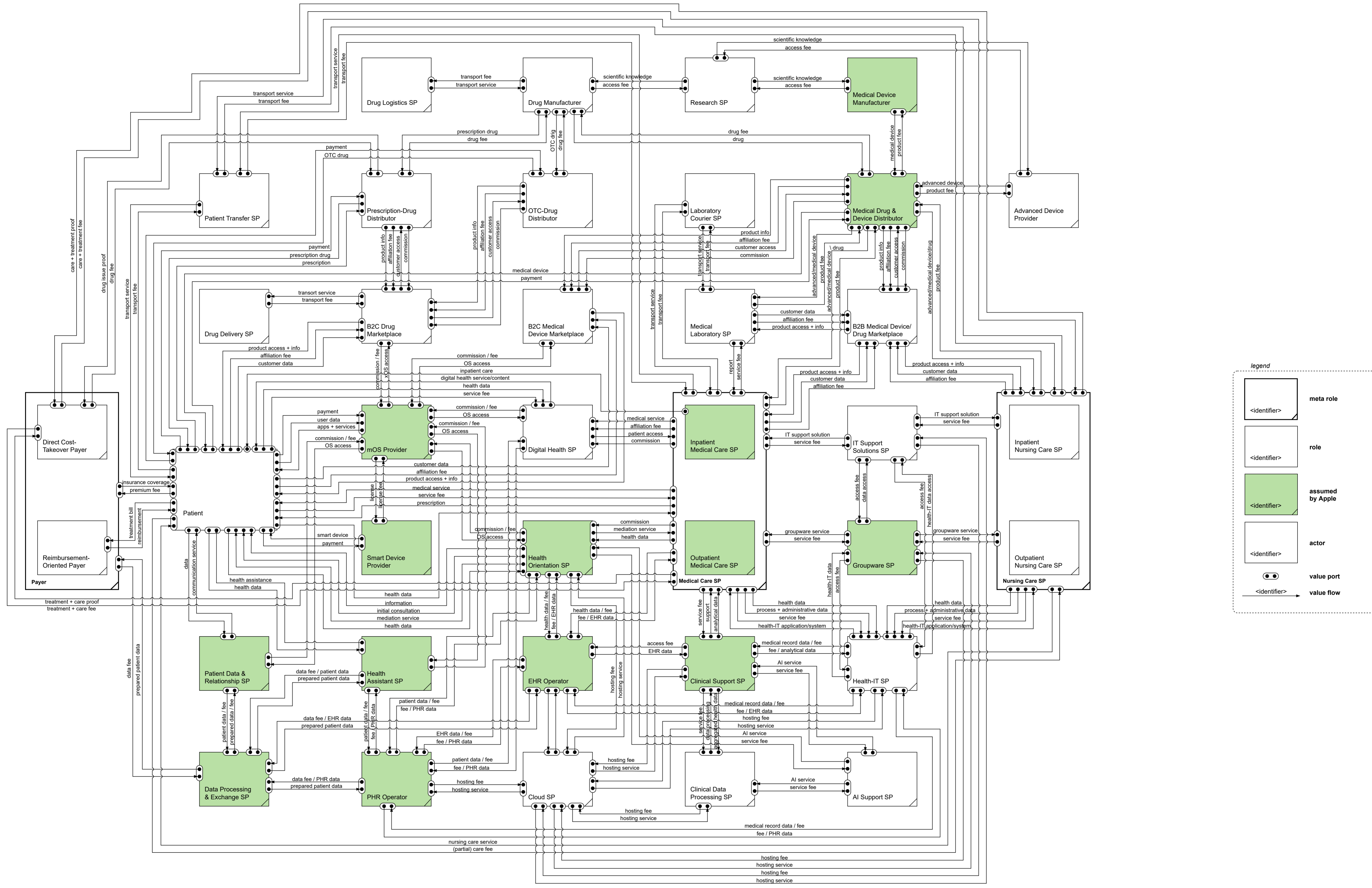

Supplement: Supplementary file 3 — (PDF 243 kb) [file 12525_2021_467_MOESM3_ESM.pdf]

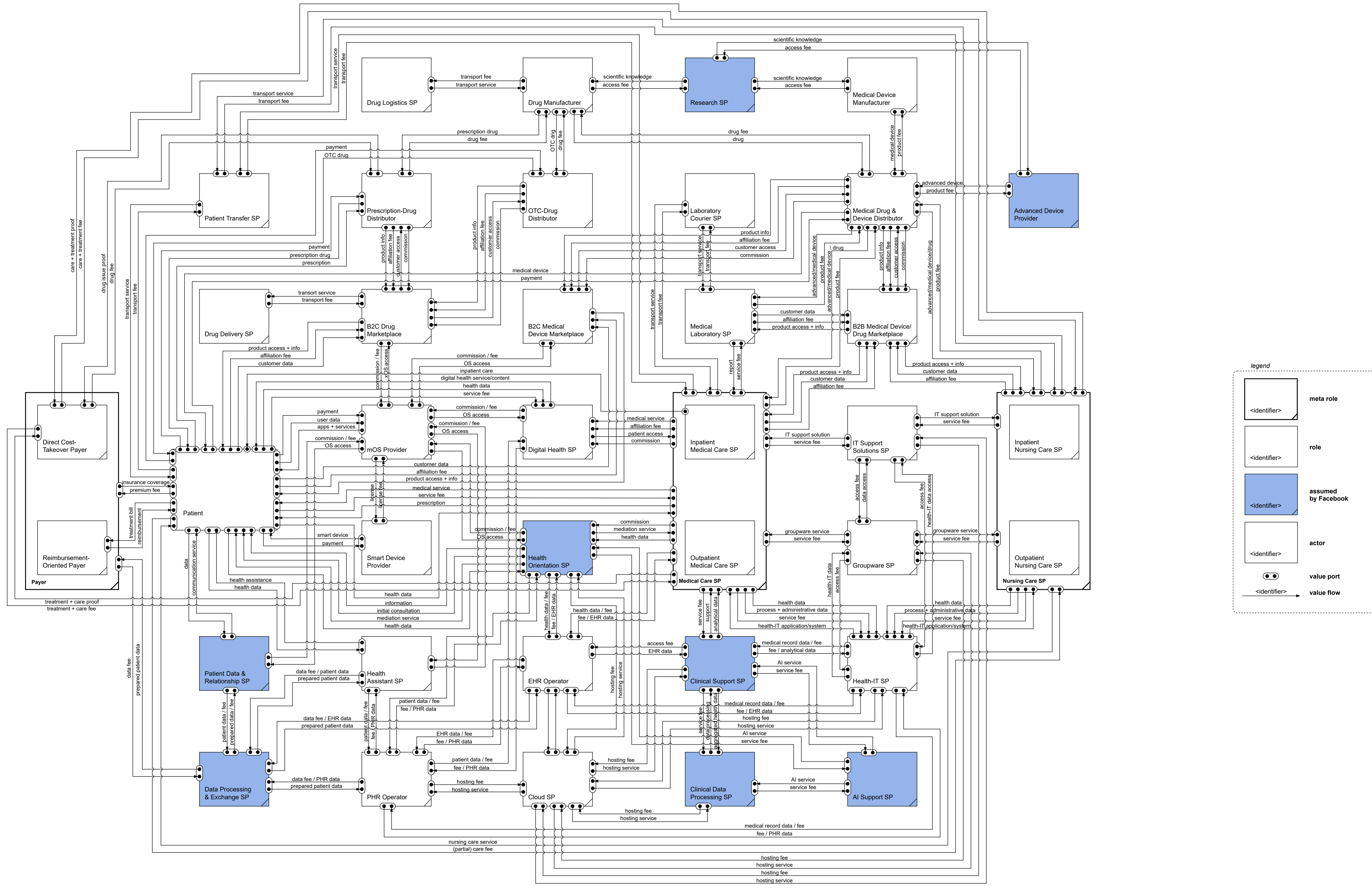

Supplement: Supplementary file 4 — (PDF 243 kb) [file 12525_2021_467_MOESM4_ESM.pdf]

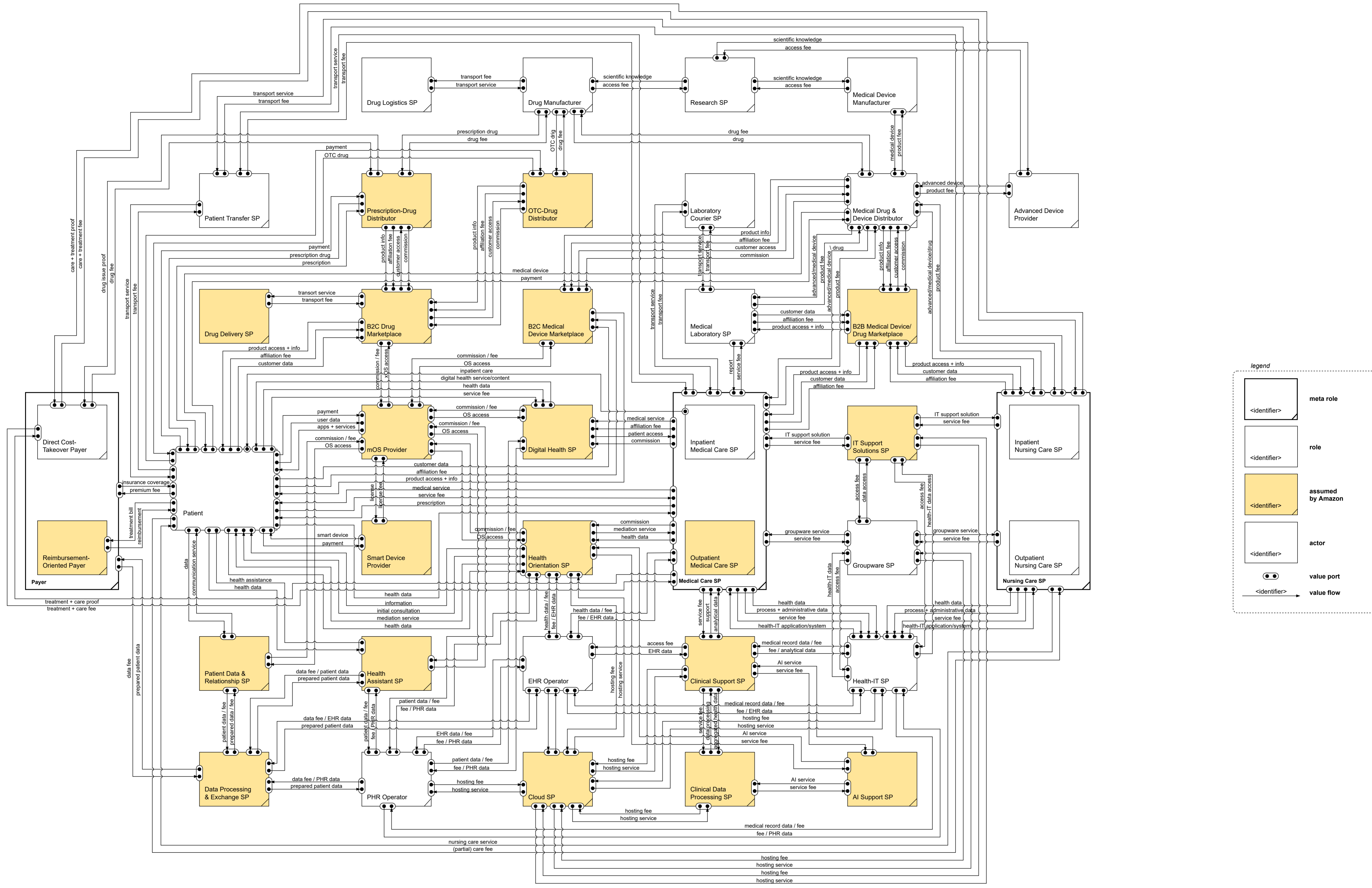

Supplement: Supplementary file 5 — (PDF 244 kb) [file 12525_2021_467_MOESM5_ESM.pdf]

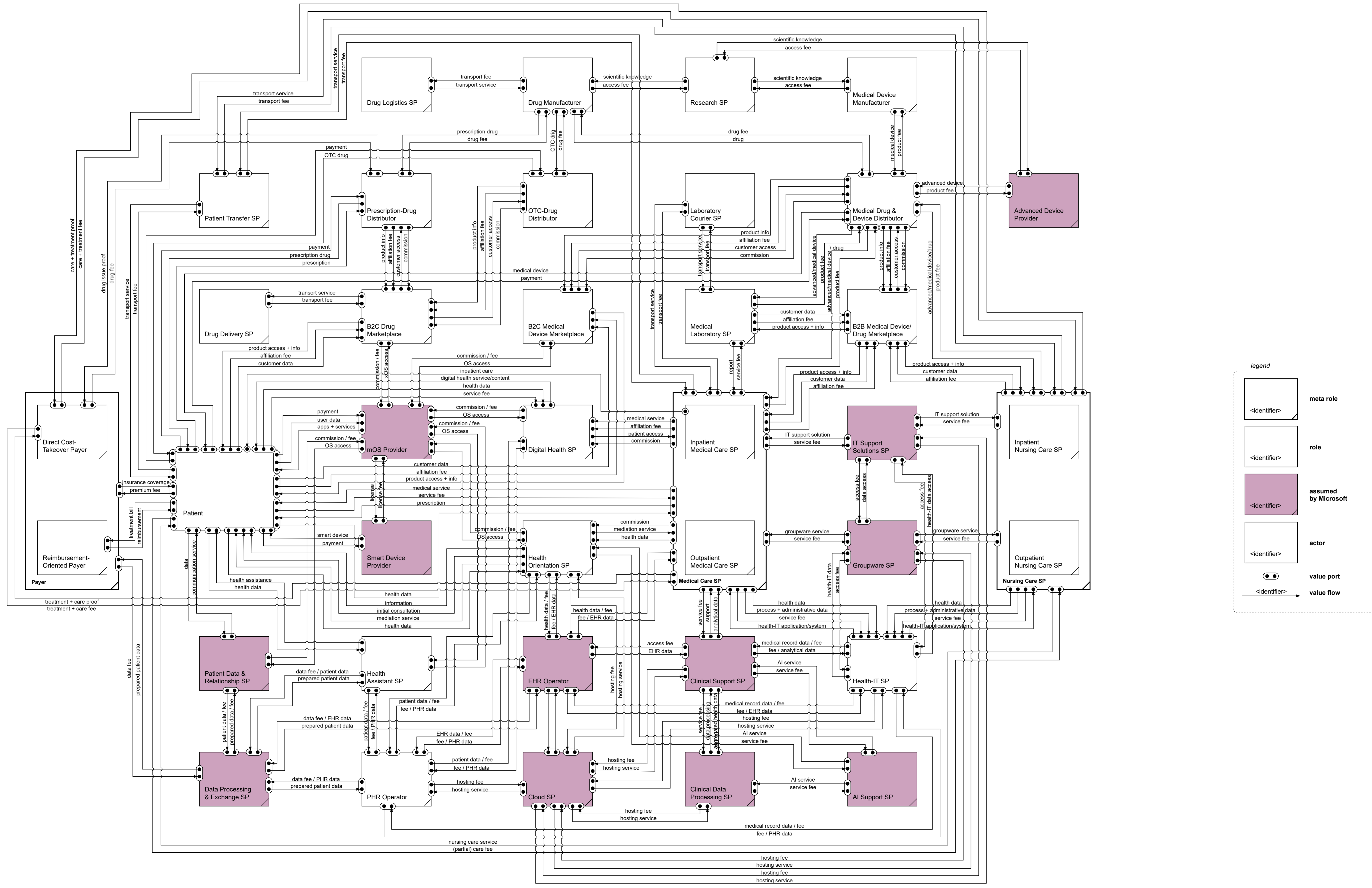

Supplement: Supplementary file 6 — (PDF 243 kb) [file 12525_2021_467_MOESM6_ESM.pdf]
